# Gut–Liver–Pancreas Axis Crosstalk in Health and Disease: From the Role of Microbial Metabolites to Innovative Microbiota Manipulating Strategies

**DOI:** 10.3390/biomedicines12071398

**Published:** 2024-06-24

**Authors:** Giada Marroncini, Laura Naldi, Serena Martinelli, Amedeo Amedei

**Affiliations:** 1 Department of Experimental and Clinical Biomedical Sciences “Mario Serio”, University of Florence, 50139 Florence, Italy; giada.marroncini@unifi.it (G.M.); laura.naldi@unifi.it (L.N.); 2 Department of Clinical and Experimental Medicine, University of Florence, 50139 Florence, Italy; 3 Network of Immunity in Infection, Malignancy and Autoimmunity (NIIMA), Universal Scientific Education and Research Network (USERN), 50139 Florence, Italy

**Keywords:** gut microbiota, hormones, metabolites, endocrine pathology, gut–liver–pancreas axis

## Abstract

The functions of the gut are closely related to those of many other organs in the human body. Indeed, the gut microbiota (GM) metabolize several nutrients and compounds that, once released in the bloodstream, can reach distant organs, thus influencing the metabolic and inflammatory tone of the host. The main microbiota-derived metabolites responsible for the modulation of endocrine responses are short-chain fatty acids (SCFAs), bile acids and glucagon-like peptide 1 (GLP-1). These molecules can (i) regulate the pancreatic hormones (insulin and glucagon), (ii) increase glycogen synthesis in the liver, and (iii) boost energy expenditure, especially in skeletal muscles and brown adipose tissue. In other words, they are critical in maintaining glucose and lipid homeostasis. In GM dysbiosis, the imbalance of microbiota-related products can affect the proper endocrine and metabolic functions, including those related to the gut–liver–pancreas axis (GLPA). In addition, the dysbiosis can contribute to the onset of some diseases such as non-alcoholic steatohepatitis (NASH)/non-alcoholic fatty liver disease (NAFLD), hepatocellular carcinoma (HCC), and type 2 diabetes (T2D). In this review, we explored the roles of the gut microbiota-derived metabolites and their involvement in onset and progression of these diseases. In addition, we detailed the main microbiota-modulating strategies that could improve the diseases’ development by restoring the healthy balance of the GLPA.

The functions of the gut are closely related to those of many other organs in the human body. Indeed, the gut microbiota (GM) metabolize several nutrients and compounds that, once released in the bloodstream, can reach distant organs, thus influencing the metabolic and inflammatory tone of the host. The main microbiota-derived metabolites responsible for the modulation of endocrine responses are short-chain fatty acids (SCFAs), bile acids and glucagon-like peptide 1 (GLP-1). These molecules can (i) regulate the pancreatic hormones (insulin and glucagon), (ii) increase glycogen synthesis in the liver, and (iii) boost energy expenditure, especially in skeletal muscles and brown adipose tissue. In other words, they are critical in maintaining glucose and lipid homeostasis. In GM dysbiosis, the imbalance of microbiota-related products can affect the proper endocrine and metabolic functions, including those related to the gut–liver–pancreas axis (GLPA). In addition, the dysbiosis can contribute to the onset of some diseases such as non-alcoholic steatohepatitis (NASH)/non-alcoholic fatty liver disease (NAFLD), hepatocellular carcinoma (HCC), and type 2 diabetes (T2D). In this review, we explored the roles of the gut microbiota-derived metabolites and their involvement in onset and progression of these diseases. In addition, we detailed the main microbiota-modulating strategies that could improve the diseases’ development by restoring the healthy balance of the GLPA.

## 1. Introduction

Humans harbor more than 10^14^ microorganisms in their bodies, most of which reside in the gastrointestinal tract, while others inhabit different anatomical sites such as the respiratory tract, skin, mammary gland, mucosa, and urogenital tract [1,2]. They form complex and distinct ecosystems, adapting to the specific resident environmental of each niche, and a symbiotic cross-talk is established between the human body and its microorganisms from birth [3]. Although every human being has a unique microbiota, its composition can vary throughout life depending on various factors such as age, lifestyle, eating habits, hormone levels, and antibiotic use [3].

The gastrointestinal tract is an interface between the external environment and internal systems, and the gut microbiota (GM) operates in symbiosis with the host, responding to external stimuli and metabolizing various substances [4]. Bioactive metabolites are a diverse array of chemicals produced by the GM and are generated by microbial degradation of various substrates (primarily dietary components) and host-derived compounds, and they influence local and systemic metabolic pathways [5]. In this scenario, further research is critical to thoroughly identifying the metabolites produced and modified by GM and exploring their functions. Using advanced metabolomics approaches, such as ultra-performance liquid chromatography and tandem mass spectrometry (LC/MS), researchers have successfully identified a wide range of metabolites which have the potential to trigger physiological and pathological functions in hosts and other bacteria, such as short-chain fatty acids (SCFAs), bile acids (BAs) and gut-derived hormones (e.g., ghrelin, glucagon-like peptide 1 (GLP-1). In this review, we explored the complexities of the GM metabolite functions in the gut–liver–pancreas axis (GLPA) crosstalk, focusing on their roles in the pathogenesis of some related disorders, including non-alcoholic steatohepatitis (NASH)/non-alcoholic fatty liver disease (NAFLD), hepatocellular carcinoma (HCC), and type 2 diabetes (T2D). Addressing NASH, NAFLD, HCC, and T2D through crosstalk of the GLPA offers a comprehensive approach to better manage these interrelated diseases. By intervening in shared pathways and mechanisms, such as inflammation, endocrine signaling, and microbiota composition, treatments can achieve more effective and lasting results, decreasing the burden of these chronic disorders.

## 2. Methods

We conducted a PubMed search for original articles, reviews, meta-analyses, and case series using the following keywords, their associations, and their acronyms: gut microbiota, gut–liver–pancreas axis, gut–liver axis, non-alcoholic steatohepatitis, non-alcoholic fatty liver disease, hepatocellular carcinoma, type 2 diabetes, glucagon-like peptide 1, bile acids, GM metabolites, fecal microbiota transplantation, short-chain fatty acids, and microbiota-changing strategies. The items found from the above-mentioned sources were reviewed by the authors, performing a narrative review. A total of 536 studies were evaluated for this revision. Of these, 218 were discarded, making for a total of 316 references in the text.

## 3. Microbiota Metabolites

The interplay between the GM metabolites is a complex network which regulates several host processes. Indeed, SCFA action in the colon can affect GLP-1 production, which in turns acts at pancreatic levels, regulating hormone release. Dysregulated the microbial metabolites’ process can contribute to various health problems, and understanding the complex relationships between GM metabolites and host health is a rapidly growing field, with potential implications for the development of targeted therapies and dietary interventions to promote health and treat diseases.

### 3.1. Short-Chain Fatty Acids and Glucagon-like Peptide-1

The SCFAs, such as acetic acid, propionic acid, and butyric acid, are molecules produced by some bacteria through the digestion, fermentation, and catabolism of indigestible dietary fibers, proteins, and glycoproteins. Acetate can be generated by enteric bacteria and acetogens, such as *Blautia hydrogenotrophica*, through the acetyl-CoA and Wood–Ljungdahl pathways, respectively. Propionate is primarily synthesized through the Firmicutes lactate pathway and the Bacteroidetes succinate pathway [6,7]. The propionate production is additionally associated with a limited number of bacterial genera, such as *Akkermansia municiphila*, that is able to degrade mucin. Butyrate instead is generated by *Firmicutes*, including *Eubacterium rectale*, *Faecalibacterium prausnitzii*, *Ruminococcus bromii*, and *Eubacterium hallii*, which are able to ferment resistant starch [8].

**Figure 1:**
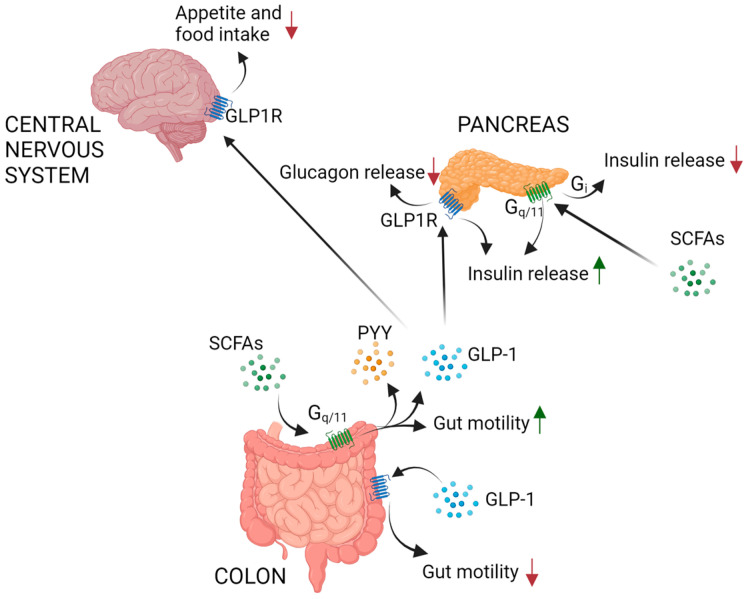
Schematic representation of SCFA and GLP-1 physiological roles. SCFA-activated colonic FFA2 triggers increased gut motility, increases or decreases insulin secretion, and stimulates the release of anorectic hormones PYY and GLP-1 from colonic crypts. GLP-1 in turn (i) decreases appetite by targeting the brain, (ii) enhances insulin secretion, (iii) suppresses glucagon secretion, (ii) slows down gastric emptying. The figure was created using Biorender.com.

A significant SCFA amount is readily metabolized by colonocytes, while the other portion is assimilated in the hepatic venous and portal systems and subsequently released from the liver into the systemic circulation [9]. SCFAs play a pivotal role in several biological processes, encompassing immune, metabolic, and intestinal effects. G-protein coupled receptors (GPCRs) and free fatty acid receptor1, free fatty acid receptor2, and free fatty acid receptor3 (FFA1, FFA2 and FFA3) constitute the primary receptor types activated by SCFAs. These receptors are expressed in various tissues and have a role in regulating both energy metabolism and immune response [10]. Metabolically, SCFAs are an abundant energy source for colonocytes, muscles, kidneys, the liver, as well as the brain and heart. Acetate and propionate can modulate energy intake by decreasing appetite through central mechanisms; Cani and colleagues observed that rats subjected to a high-fat diet (HFD) enriched with fermentable carbohydrates (FCs) exhibited a reduced dietary energy intake, adipose tissue presence, and body weight [11]. Moreover, administration of butyrate and propionate to rodents decreased obesity and improved sensitivity to insulin and energy expenditure [12,13]. These data demonstrated that SCFAs can protect against obesity induced by diet and can control gut hormones. In addition, elevated intake of dietary FCs contributes to normalizing appetite control and the regulation of body weight [11]. Frost and colleagues documented that FC-derived acetate can suppress appetite by acting directly on central hypothalamic mechanisms, including alterations in transcellular neurotransmitter cycles [14]. Indeed, FCs are believed to stimulate the release of anorectic gut hormones, including peptide YY (PYY) and GLP-1 [15,16,17]. The SCFA mechanism to induce the release of these anorectic hormones is the activation of FFA2 in enteroendocrine cells, thus facilitating a signaling cascade involving Gq/11, inositol phosphate, and calcium [15,18,19]. Pancreatic β cells express SCFA receptors even if their role is not clearly elucidated. A regulatory relationship between SCFAs and the FFA2 in insulin secretion was shown by reduced insulin and elevated plasma glucose levels in FFA2 knockout (KO) embryos compared to the wild type [20]. Regarding FFA3, evidence from transgenic mouse models suggest a potentially inhibiting impact of this receptor on insulin release. The different effects can be explained by the different signaling pathways downstream of SCFA receptors. Signaling through FFA3 in pancreatic β cells is associated with Gi proteins that exert an inhibitory effect on insulin secretion, while FFA2 can also activate Gq /11 signaling, leading to promotion of insulin secretion [19,21] (Figure 1). Additionally, SCFAs protect the integrity of the gut epithelium by competitively inhibiting the growth of pathogenic bacteria and preventing the permeability lack of the gut barrier, thus protecting from metabolic endotoxemia associated with obesity, adipose inflammation, and leaky gut-derived insulin resistance (IR) [22,23].

GLP-1 peptide is produced by the L-cells of the small intestine and proximal colon in direct response to luminal molecules, such as SCFAs, and it serves as an incretin (Figure 1). GLP-1 binds to its receptor glucagon-like peptide-1 receptor 1 (GLP-1R), which is a G-protein coupled receptor (GPCR) present in several tissues, including the brain and pancreas. Its main effect is lowering gastrointestinal motility, delaying gastric emptying, and enhancing satiety [24]. Within the brain, GLP-1 impacting in the hypothalamus and brainstem decreases food consumption and body weight [25]. Regarding its role in the pancreas, GLP-1 enhances insulin secretion from β cells in the presence of hyperglycemia and concomitantly suppresses the release of glucagon from α cells [26] (Figure 1).

### 3.2. Bile Acids

**Figure 2:**
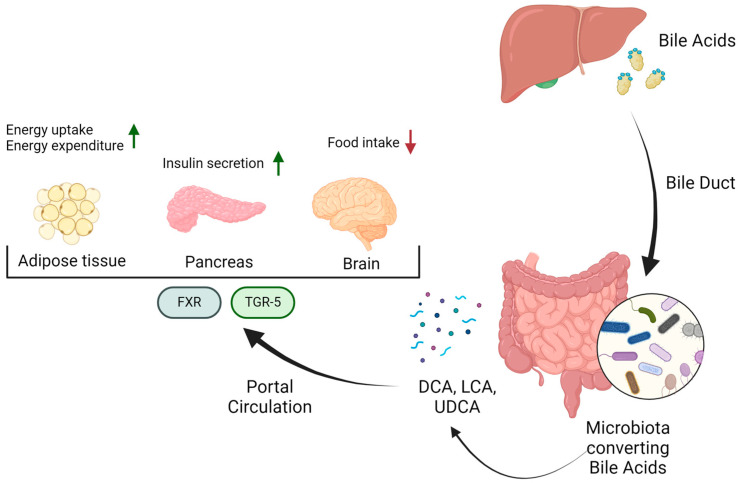
Schematic representation of BA metabolism. BAs are produced by hepatocytes, stored in the gallbladder, and secreted. Subsequently, GM can metabolize BAs in secondary BAs (DCA, LCA, UDCA), which regulate the metabolism of adipose tissue, pancreas, and brain through FXR and TGR-5 signaling pathways by increasing energy uptake and insulin secretion and decreasing food intake. The figure was created using Biorender.com.

One of the most important classes of GM metabolites are bile acids (BAs), amphipathic molecules synthesized by hepatocytes and derived from cholesterol. BAs are stored in the gallbladder and are released into the small intestine in response to cholecystokinin triggered by a meal [27]. In the small intestine, they solubilize dietary lipids by forming micelles in the small intestine and improve the absorption of lipids, sterols, and vitamins. Subsequently, they are reabsorbed in the terminal ileum and transported back to the liver through the portal circulation. The relationship between BAs and the GM involves bilateral interactions, since BAs and microbiota reciprocally control their composition. Indeed, GM can metabolize BAs by influencing the expression of some key enzymes involved in de novo BA synthesis [28]. The transformations exerted by the GM include deconjugation, dehydroxylation, re-conjugation, epimerization, oxidation, and other reactions [28,29,30]. BAs can consequently bind to their receptors, such as the farnesoid X receptor (FXR), G-protein-coupled receptor-1 (TGR5/GPBAR1), pregnane X receptor (PXR), and vitamin D receptor (VDR), thus modulating metabolic and immunological functions within the host [31]. Primary BAs, such as cholic acid and chenodeoxycholic acid (CDCA), are converted into secondary BAs, deoxycholic acid (DCA), lithocholic acid (LCA) and ursodeoxycholic acid (UDCA), respectively, solely by GM BA-inducible operon (BAi-operon) via the 7-α-dehydroxylation process, by species such as *Lactobacillus*, *Bacteroides*, *Bifidobacterium*, *Clostridium*, *Eubacterium*, *Enterococcus* [32,33,34,35]. Finally, the GM can also esterify BAs, making them more hydrophobic [36]. BAs can activate FXR and TGR-5 signaling pathways, thus suppressing gluconeogenesis and increasing glycogen synthesis in the liver. This activation enhances glucose-stimulated insulin release in the pancreas and boosts energy expenditure, especially in skeletal muscles and brown adipose tissue. Moreover, in the brain, the signaling through TGR5 mediates satiety [37,38,39] (Figure 2). Additionally, BAs can influence the GM composition directly by disrupting bacterial membrane structures and indirectly by activating FXR, leading to the production of antimicrobial agents such as Inducible Nitric Oxide Synthase (iNOS) and interleukin-18 (IL-18) in the small intestine, consequently hindering bacterial growth [40,41]. Several compounds, such as plant or animal extracts, can modulate GM, BA metabolism, and the associated signaling pathways [42,43]. For example, grape seed proanthocyanidin and polyphenol extracts from pomegranate have been shown to enhance LCA production [44,45]. When an imbalance in the GM communities occurs, dysbiosis is established. Dysbiosis can induce symptoms such as digestive problems (e.g., abdominal bloating, gas, diarrhea), changes in bowel habits, and fatigue. This phenomenon can be caused by various factors, such as age, diet, stress, infection, and antibiotic use [46,47,48]. Since the GM influences several physiological processes, such as regulation of metabolism, its alteration can result in a whole range of undesirable effects, including metabolic problems and the establishment of chronic diseases [49]. Because of its implication in these different processes, recent evidence has shown that GM alterations promote the development of several neoplasms, including lung cancer, gastrointestinal cancer and HCC, as well as pancreatic cancer (PC) [50,51,52,53].

## 4. Microbiota Metabolites and Gut–Liver Axis

The interaction between microbiota metabolites and the GLPA is gaining increasing attention due to the profound impact of these metabolites on physiological functions and their role in the development of various diseases. Understanding the complex crosstalk between microbiota metabolites and the GLPA is essential for unraveling the mechanisms of various diseases, including obesity, diabetes, NAFLD, pancreatic disorders, and, not least, cancer.

T2D, obesity, and NAFLD are associated with dysbiosis and changes in SCFAs, and the pool size of BAs is able to trigger an imbalance between the physiologic and pathologic condition [54,55]. Indeed, a healthy GM maintains a symbiotic relationship with the host, but when quantitative and qualitative modifications occur, the development of a chronic disease could be promoted. The intestine and the liver are strictly connected through the portal vein and the biliary tract. GM alterations can induce tight-junction disassembling and increase intestinal permeability, allowing the harmful microbiota metabolites to reach the liver by portal circulation [56]. As a consequence, the microbiota endotoxins penetrate the portal vein and decrease the release of a fasting-induced adipose factor (FIAF) by increasing lipoprotein lipase (LPL) activity, promoting de novo fatty acid synthesis and triglyceride production and activating inflammatory TLRs in hepatocytes [57]. In turn, TLRs on the hepatocyte surface interact with gut hormones and metabolites, promoting steatosis, inflammation fibrosis, and IR. In detail, TLR4 is expressed on the plasma membrane of hepatocytes and Kupffer cells, and TLR4-mediated signals are thought to activate signaling molecules such as nuclear factor kappa B (NFkB), which induces the production of inflammatory cytokines (IL-1β and IL-18) and liver injury [58]. The persistent exposure of TLRs to gut derived metabolites alters hepatic immunotolerance and establishes a local low-grade inflammation that concurs to NAFLD/NASH progression [59]. The fat accumulation in the liver, a condition known as simple steatosis, is usually considered the first “hit” in this pathological progression. Over time, continued lipid synthesis and uptake in the liver contribute to increased oxidative stress and inflammation. Indeed, NASH is the result of multiple factors acting simultaneously, including genetic variants, abnormal lipid metabolism, oxidative stress, altered immune response, and GM imbalances [60]. This persistent damage triggers fibrosis, a process characterized by the accumulation of scar tissue in the liver that, finally, can lead to liver damage and dysfunction [61,62]. Sometimes NASH can progress to liver cirrhosis and HCC. Moreover, the NAFLD severity and its progression to NASH has been associated with GM dysbiosis and loss of commensal bacterial metabolic functions [63]. In NAFLD, changed GM composition has been observed at various taxonomic levels. Specifically, there is a reported decrease in the abundance of Bacteroidetes at the phylum level, while Firmicutes and Proteobacteria show increased levels [64,65,66]. At the family level, *Enterobacteriaceae* has been found to be increased, whereas *Rikenellaceae* and *Ruminococcaceae* are decreased [63,67]. Therefore, many GM metabolites, mainly SCFAs, BAs and gut hormones as glucose-dependent insulinotropic peptide (GIP) and incretin GLP-1, have been associated with liver diseases [68].

### 4.1. SCFA Roles in NAFLD/NASH

As mentioned above, dietary soluble fibers are fermented by gut bacteria into SCFA, which are generally considered to be health promoting. Although butyrate is predominantly used by the gut epithelium and propionate is primarily metabolized in the liver, the acetate attains the highest concentrations in plasma among the SCFAs [69]. Following absorption, acetate exhibits the highest abundance in the liver and achieves the highest peripheral concentrations (ranging from 19 to 160 μM) compared to propionate (1–13 μM) and butyrate (1–12 μM), as documented through human studies [70,71,72]. Acetate preferentially activates its receptor FFAR2 in vitro; propionate displays a similar agonist on FFAR2 and FFAR3; and butyrate preferentially activates FFAR3 [10,73]. On the other hand, FFAR2 activation inhibits adipocyte differentiation and increases hepatic lipogenesis, thereby promoting the NAFLD development [74]. In turn, butyrate is capable of upregulating GLP-1R expression to improve NAFLD [75].

The peroxisome proliferator-activated receptors (PPARs) are members of the nuclear receptor superfamily, and they are known for their crucial roles in maintaining metabolic homeostasis. Three isoforms of PPARs have been identified: PPARα, PPARβ/δ, and PPARγ. These receptors function as sensors for fatty acids and play pivotal roles in regulating the various pathways involved in lipid and glucose metabolism as well as energy homeostasis [76]. PPARs have been extensively studied for their involvement in liver injury and have been identified as effective targets for the treatment of metabolic syndrome, including dyslipidemia, diabetes mellitus, and, notably, NAFLD [77,78]. The SCFA-producing bacteria may play a critical role in NAFLD [79], as documented in numerous studies, highlighting their therapeutic potential in managing metabolic disorders and even modulating PPARs signaling pathways. Indeed, in situations where there is a reduced presence of butyrate in the gut lumen, PPARγ activity tends to decrease. This decrease in PPARγ activity is linked to elevated levels of glycolysis and reduced oxygen consumption, leading to altered metabolic states. Conversely, butyrate binding to PPARγ can inhibit the expression of iNOS, resulting in decreased production of nitric oxide (NO) and, subsequently, nitrate [80]. This interplay highlights the relevant role of butyrate in modulating metabolic pathways and its potential impact on metabolic dysfunction in pathological conditions. In addition, SCFAs can activate PPARα expression and thus prevent lipid accumulation in the liver via the AMPK pathway [81,82]. There is also the stimulation of gut hormones, as GLP1, GIP, and food intake inhibition by SCFAs may represent a novel mechanism by which GM regulates the pathological progression of NAFLD to NASH [12]. Multiple studies have suggested a potential link between high SCFA levels and obesity as well as excessive fat deposition [83]. Accordingly, the oral administration of propionate and butyrate significantly reduces the severity of weight gain and IR induced by an HFD [12,84]. Moreover, oral supplementation of butyrate has been shown to ameliorate hepatic inflammation by inhibiting the TLR4 signaling pathway and the production of inflammatory cytokines such as IL-6 and tumor necrosis factor alpha (TNF-α) [85,86]. The direct effects of acetate, propionate, butyrate, and sodium acetate (NaA) on alleviating NASH phenotype was confirmed also in a mouse model of NASH. In detail, NaA was seen as being capable of decreasing hepatocyte fat deposition and suppressing macrophage pro-inflammatory responses [87]. The inhibitory effects of butyrate on multiple pro-inflammatory signaling pathways through both HDAC-dependent and -independent manners were also well characterized [88]. These findings underscore the potential SCFA role in mitigating factors contributing to NASH development. The alleviation SCFA effects on NAFLD are well established in both human investigations and animal studies. In summary, SCFAs are able to decrease liver steatosis and the progression to NAFLD/NASH, not only directly interacting with the liver but also decreasing fat accumulation in the adipose tissue, increasing the intestinal barrier function, and inhibiting gut motility, thereby enhancing nutrient absorption.

### 4.2. GLP-1 Effects on NAFLD/NASH

It is currently understood that the intestine functions as an endocrine organ capable of secreting hormones that play crucial roles in regulating whole-body metabolism in response to food intake, as well as controlling appetite and gastric emptying. Among these hormones, the most extensively studied are incretin hormones such as GLP-1 and GIP, which are primarily secreted by the intestine following a meal. Considering the low GLP-1R expression in the liver, the potential action mechanism of GLP-1R agonists (GLP-1RA) in NASH might be associated with their indirect positive effects on body weight, IR, and the mitigation of metabolic dysfunction, as well as their ability to counteract lipotoxic effects and inflammation [89]. Indeed, there is substantial debate regarding the potential GLP-1 effects on adipose tissue, muscle tissue, and the liver, compounded by uncertainty regarding the expression of the GLP-1R in these tissues. While some studies have yielded negative results regarding GLP-1R expression [90], others have provided ambiguous findings [91]. Furthermore, there may be inter-species differences, with evidence suggesting that mice may exhibit GLP-1R expression in the liver [91]. Nevertheless, the incretin hormone GLP-1 and its analogs have garnered interest in the NASH context due to their potential therapeutic effects on metabolic dysfunction and liver pathology. Indeed, obesity and T2D represent two of the major risk factors for NASH [92] and IR in the liver, and adipose tissue is a crucial driver of NASH morbidity and mortality [93]. The mechanisms through which gut hormones regulate hepatic lipid metabolism are still unclear. However, in vitro studies have demonstrated that GLP-1-treated hepatocytes manifested a significant increase in cAMP production and a reduction in mRNA expression of stearoyl-CoA desaturase 1 and genes associated with fatty acid synthesis, such as acetyl-CoA carboxylase and fatty acid synthase [94,95]. Moreover, the release of GLP-1 in vitro and in vivo is due to SCFA derived GM metabolites, and GLP-1 can stimulate dependently of the FFAR 2 the progression of steatosis to NASH, as already mentioned [15]. Thereafter, in the HFD-fed mouse model, hepatic GLP-1 levels were decreased, and sodium butyrate was reported to increase the expression of GLP-1R [96]. Although the Food and Drug Administration (FDA) did not approve GLP-1RA for the treatment of NAFLD and NASH, diseases whose rates are increasing worldwide, even more data underline their hepatic therapeutic effects. Indeed, in the liver, incretins primarily regulate glucose homeostasis by influencing insulin and glucagon secretion. Indeed, GLP-1RAs have demonstrated beneficial effects in preclinical models and clinical studies of NASH through the promotion of weight loss and the decrease in liver fat [97]. The impact of GLP-1RAs on hepatic steatosis has been observed in various mouse models, including leptin-deficient ob/ob mice, leptin receptor-deficient db/db mice, and mice fed an HFD. Treatment with exenatide or liraglutide, incretin mimetics drugs, has been shown to result in a reduction in hepatic steatosis in these models [94,98]. Generally, GLP-1RAs have been associated with a decrease in hepatic triglyceride (TG) and with an improvement in liver enzymes, particularly noticeable in patients with elevated levels of alanine aminotransferase (ALT) at the beginning of treatment [99]. The LEAN (Liraglutide Efficacy and Action in NASH) trial stands as the largest study to date assessing the effectiveness of liraglutide, a GLP-1RA, in individuals with biopsy-proven NASH. In this trial, fifty-two patients were randomly assigned in a 1:1 ratio to receive either subcutaneous injection of liraglutide or a placebo. The results indicated that liraglutide led to the NASH resolution in 39% of patients compared to 9% in the placebo group [100]. In this study, fewer patients in the liraglutide group (2/23) showed progression in fibrosis compared to the placebo group (8/22), and a greater proportion of liraglutide-treated patients had improved steatosis and hepatocyte ballooning; the improvement was associated with weight loss and a marked reduction in aspartate transaminase (AST) and ALT levels.

### 4.3. BAs and their Role in NASH

It has been shown that, in NAFLD, the abundance of bacteria that convert primary BAs into secondary BAs is decreased [101]. In the NAFLD pathogenesis, the signaling pathways activated by BAs, which mainly involve FXR and TGR5, are under-stimulated as DCA is decreased [102]. Therefore, FXR and TGR5 regulate multiple signaling pathways, maintaining glucose homeostasis, reducing hepatic steatosis, and promoting anti-inflammatory responses [103]. Accordingly, KO FXR mice showed elevated serum BAs, increased serum and hepatic cholesterol and TGs, and a pro-atherogenic serum lipoprotein profile [104], correlating with the NAFLD development [105]. Analogously, in a mouse model of hypercholesterolemia, FXR deficiency promotes the pathological progression to NASH [106]. On the other hand, FXR activation may reduce NAFLD inhibiting lipogenesis, hepatic inflammation, and fibrosis in rats, maintaining intestinal barrier integrity and thus protecting the liver from bacteria-derived inflammatory signals [40,107]. Hepatic FXR activation in NASH exerts systemic effects that include the upregulation of fatty acid oxidation, inhibition of de novo lipogenesis and fatty acid synthesis via SHP-mediated suppression of SREBP-1c, and decreased gluconeogenesis, thereby mitigating IR. Additionally, FXR activation suppresses inflammation by inhibiting NF-κB, nucleotide-binding domains, leucine-rich–containing families, pyrin domain–containing-3 (NLRP3), and chemokines C–C motif chemokine ligand 2 (CCL-2), and it decreases fibrosis by inhibiting transforming growth factor β1 (TGF-β1) secretion and extracellular matrix deposition by hepatic stellate cells (HSCs). Furthermore, FXR activation enhances the production of fibroblast growth factor 21 (FGF21), resulting in decreased cholesterol and TG synthesis, lower glucose levels, increased adiponectin expression, induction of adipocyte browning, and further attenuation of liver inflammation and fibrosis [108,109,110,111]. On the other hand, TGR5 agonists have been shown to decrease liver steatosis and associated hepatocyte damage, as evidenced by reductions in plasma liver enzymes LDH, ASAT, and ALAT in an HFD mouse model [112]. This improvement is accompanied by decreased levels of plasma TG and non-esterified fatty acids. Consistently, male TGR5 KO (TGR5−/−) fed an HFD for 8 weeks exhibited pronounced hepatosteatosis [113]. These findings suggest that TGR5 activation may have an anti-NAFLD protective effect. However, the right mechanisms by which TGR5 exerts this effect have yet to be fully elucidated. Although TGR5 expression is not detected in hepatocytes, it is present in various liver cell types, directly or indirectly influencing liver function and triglyceride metabolism. Notably, TGR5 is enriched in Kupffer cells, resident liver macrophages, which can secrete pro-inflammatory cytokines and contribute to NAFLD progression. Additionally, TGR5 has been implicated in modulating microcirculation and fluid secretion in endothelial and biliary epithelial cells within the liver [114,115]. Although FXR is the master regulator of BAs, PPARs can also modulate their metabolism. In hepatocyte, PPARα is a target gene of FXR [116]. During liver inflammation, the activation of the NF-κB pathway leads to the inhibition of hepatic FXR. This inhibition suppresses BA detoxification by reducing the expression of PPARα and downstream enzymes, such as cytochrome P450 enzymes (CYPs), sulfotransferases (SULTs), and UDP-glucuronosyltransferases (UGTs). These enzymes are responsible for the Ba detoxification. This dysregulation of BA detoxification contributes to the pathogenesis of liver inflammation and associated disorders [117].

Overall, understanding the complex interactions between BAs and the GM is crucial for unraveling the mechanisms underlying NASH pathogenesis and developing novel therapeutic interventions for this increasingly prevalent liver disease.

## 5. GM Derived Metabolites and Gut–Pancreas Axis

Considering the anatomical proximity of the pancreas to the gastrointestinal tract, there is a potential mechanism where the microbiota from the esophagus, stomach, duodenum, or biliary tract may enter the pancreatic parenchyma through the pancreatic duct. Another potential method of bacterial introduction into the pancreas could involve translocation from the gut. This explanation is anatomically plausible, as mesenteric venous drainage passes near the pancreas on its way to the liver. T2D patients showed an increase in multiple pathogenic bacteria, such as Clostridium hathewayi, Clostridium symbiosum and Escherichia coli. Conversely, healthy controls (HC) exhibited a higher abundance of bacteria known for producing butyrate [118]. Metabolites as BAs, SCFAs, trimethylamine (TMA), and incretins produced by GM have the potential to impact the T2D progression influencing various physiological mechanisms, including β cell dysfunction, chronic low-grade inflammation, oxidative stress, and dysregulation of lipid and glucose metabolisms.

### 5.1. SCFA Involvement in T2D

For instance, SCFAs have been shown to decrease the expression of pro-inflammatory cytokines by inhibiting NF-κB activation and the inhibiting of nuclear factor kappa B (IκBα) degradation. This, in turn, can enhance glucose regulation and attenuate the T2D development. Acetate, one of the most abundant SCFAs, acts by binding to the G-protein coupled receptors (GPR), GPR43 (FFAR2), and GPR41 (FFAR3) that have been shown to be expressed at the mRNA level in various insulin sensitive tissues, such as the adipose tissue [73], liver [119] and pancreatic β cells [21,120]. Indeed, both murine and human β cells express GPRs (FFA2, FFA3), through which acetate has been reported to modulate insulin secretion [21]. Supporting this, mice deficient in FFAR2 show glucose intolerance as well as decreased β cell mass and function [121]. In addition, acetate at physiological concentrations (70–170 μmol/L) [9] only increased in vitro insulin secretion in a murine β cell line and not in human pancreatic islets, suggesting a species-specific differences [21]. Nevertheless, acetate may potentially modulate circulating levels of insulin, affecting insulin secretion directly via G protein-mediated signaling or indirectly via vagal/parasympathetic activation [122]. SCFA receptors FFA2 and FFA3 have been viewed as potentially attractive drug targets for the regulation of metabolic and related disorders [19] thanks to their relationship to the endocrine, metabolic, and inflammatory systems. Specifically, the SCFA-FFA2 axis seems to regulate insulin secretion, as evidenced by the reduced insulin levels and elevated plasma glucose levels observed in FFA2 knockout embryos compared to wild-type embryos [20]. Regarding FFA3, findings from transgenic mouse models suggest a negative impact of this receptor on insulin release. Similarly, in an in vivo glucose tolerance test, FFA3 KO mice showed lower glucose levels than the wild type, whereas the opposite was observed in FFA3-overexpressing mice [123,124]. A study using the homeostatic model of IR assessment (HOMA-IR) observed an adverse correlation between blood insulin levels and total SCFAs, including acetate and propionate [125], while in vivo studies showed that diabetic rodents exhibited lower levels of acetate, propionate, and butyrate compared with controls [126]. For these reasons, there is interest about the beneficial effects of SCFAs in T2D.

In vitro and in vivo studies have shown that propionate can increase insulin release, support the presence of β cells by decreasing trans-differentiation in αcells, hinder apoptosis, and promote proliferation [127]. In T2D, propionate is also involved in the downregulation of chemokines and inflammatory cytokines, such as CC chemokine ligand-5 (CCL-5) and TNF-α. Similarly, butyrate has the ability to improve insulin sensitivity [128]. In a model of prediabetic mice, butyrate treatment suppressed obesity and IR [129]. In agreement, in a cohort of 100 subjects, Li. et al. observed that the risk of developing T2D was significantly lower in subjects with butyric acid levels > 585.031 μg/g [130].

### 5.2. GLP-1 Incretin and T2D

The intestine is also responsible for the secretion of GLP-1 and GIP upon nutrient stimulation, which go through the liver, reach circulation, and target the pancreas to regulate insulin and glucagon secretion. Pancreatic GLP-1 stimulates glucose-dependent insulin secretion by β cells and downregulates secretion of glucagon by α cells [131], conferring glucose sensitivity to glucose-resistant β cells; it also stimulates β cell proliferation and neogenesis and inhibits β cell apoptosis. As already mentioned, the GM is involved in obesity, NAFLD, IR, and chronic inflammation, which are related to the T2D development [132,133], and GLP-1RA have been shown to reduce hyperlipidaemia, hypertension, and fatty liver in diabetic patients [134]. Interestingly, TGR5 activation by microbiota metabolites in enteroendocrine L-cells increases the secretion of GLP-1. Then, the GLP1/GLP-1R binding in pancreatic β cells increases insulin secretion and reduces glucagon synthesis, and this is why GLP-1RA have been proposed as antidiabetic drugs. Notably, evidence has demonstrated the efficacy and safety of GLP-1RA for the treatment of T2D. GLP-1-GIP dual agonists (tirzepatide), GLP-glucagon receptor dual agonists (cotadutide), or triple agonists against GLP-1-GIP-glucagon receptors have gained much attention as novel hypoglycemic agents capable of better controlling blood glucose levels and body weight, improving hepatic lipid metabolism and systemic insulin sensitivity [135]. Importantly, in May 2022, the US FDA approved tirzepatide as the first dual GLP-1 and GIP receptor agonists for the T2D treatment [136]. Due to their numerous effects, dual incretin receptor agonists could also represent a potential treatment for patients with MAFLD [136].

Finally, recent items discuss the potential use of metformin in T2D patients. Metformin administration, through a mechanism of action that involves incretins (mainly GLP-1 [137]), induces the reduction in hepatic glucose production, primarily by decreasing gluconeogenesis. Indeed, metformin increases GLP-1 levels, and it may also impact the expression of GLP-1R in pancreatic β cells. These effects on incretin signaling pathways are both independent/dependent on PPARα [138].

### 5.3. BAs Roles in T2D

As previously mentioned, the GM can also influence the activity of FXR and TGR5 through altering the composition of the BA pool [41]. It has been found that GM dysbiosis can result in reduced production of secondary BAs and diminished activation of BA receptors. This disruption in BA signaling further contributes to dysregulated glucose metabolism and the development of T2D, mainly regulating carbohydrates, lipids, and energy metabolism [139,140]. While levels of peripheral blood BAs do not serve as predictors for the transition from impaired fasting glucose (IFG) to new-onset diabetes (NOD) [141] in preclinical animal models of diabetes, a non-absorbable polymeric BA chelator (SAR442357) has been reported to improve hyperglycemia [142]. Moreover, BAs regulate the GM composition by activating TGR5/FXR receptors, which maintains the stability of intestinal environment and, in turn, improves T2D [143]. The activation of the FXR enhance lipid metabolism and improve insulin sensitivity in obese Zucke (fa/fa) rats, ob/ob mice, and db/db diabetic mice [144,145]. Furthermore, Bingting Chen et al. have recently found that glycoursodeoxycholic acid (GUDCA), a glycine-conjugated form of the secondary BA UDCA, positively regulates the GM by altering BA metabolism and effectively improves oxidative stress damage, reducing IR in a in db/db mice after continuous administration [146]. Many clinical reports demonstrate that high circulating levels of BAs had beneficial effects on glucose metabolism, improved insulin sensitivity, and induced better postprandial glycemic control [147,148]; therefore, BAs regulate glucose homeostasis through activation of TGR5 [149]. In agreement, in vitro studies demonstrated that TGR5 induces GLP-1 secretion in cultured mouse enteroendocrine STC-1 cells [150] and that the semisynthetic BA, 6-ethyl-23(S) methylcholic acid (6EMCA or INT-777), which is a specific TGR5 agonist, induces GLP-1 secretion in both STC-1 cells as well as in human intestinal NCI-H716 cells, also showing that it contributes to glucose homeostasis. On the other hand, the knock down of TGR5 in STC-1 cells prevented the secretion of GLP-1. Regarding FXR agonists, the semisynthetic compound INT-747 (6-ethyl-CDCA or obeticolic acid) exerts a hepatoprotective effect in fibrosis [151], and protects against IR and hepatic steatosis [145]. In addition, INT-747 treatment was shown to result in small (1–2%), but significant, weight loss and improved insulin sensitivity in patients with NAFLD [152].

These clinical reports suggest that GM and its metabolites may be significantly associated with T2D progression [153], and GLP-1R and FXR agonists have been proposed as a viable pharmaceutical strategy for treating diet-induced T2D.

## 6. Gut–Liver–Pancreas Axis: A Critical Metabolic Crosstalk

Microbiota metabolites have a central role in the interaction between the gut, liver and pancreas in terms of control of metabolism and inflammation in obesity, dyslipidemia, and NAFLD. The concept of the gut–liver–pancreas axis has become increasingly significant, particularly with evidence suggesting that its dysregulation can contribute to the development of NAFLD and potentially predispose individuals to NASH. Furthermore, various therapeutic interventions aimed at modulating the GPLA, such as incretin-based therapies, have demonstrated encouraging outcomes in the treatment of NASH. Furthermore, GM composition and dietary habits are associated with an increased likelihood of obesity. The excessive FFAs released from both microbiota and adipose tissue induce a pro-inflammatory state characterized by an excessive reactive oxygen species (ROS) and pro-inflammatory cytokines released into the bloodstream. The surplus FFAs and dietary lipids infiltrate non-adipose organs like the liver and pancreas, resulting in ectopic fat deposition and lipotoxicity, causing a prolonged low-grade systemic inflammation. These conditions additionally interfere with insulin action in the insulin signaling pathway, perturb glucose homeostasis, and foster systemic dysregulation.

**Figure 3:**
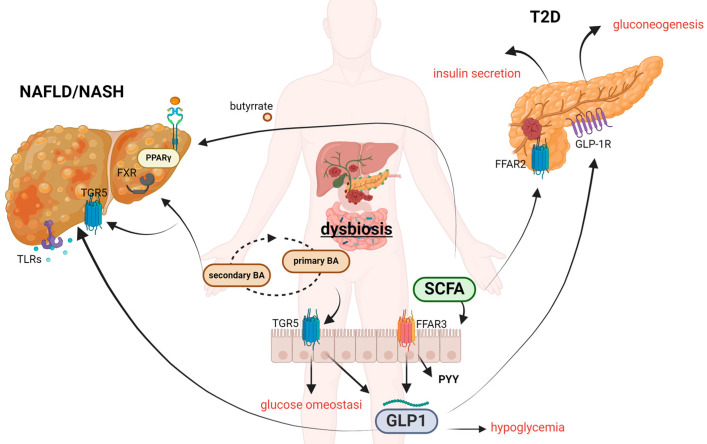
Interplay of gut, liver and pancreas in metabolic NASH and T2D disease. Dysbiosis is a condition due to GM alterations that plays a crucial role in liver and pancreas disease. The increase in imbalance between primary/secondary BA production induces the activation of intestinal TGR5, which contributes to increasing serum GLP-1 pool, and of hepatic TGR5 and FXR involved in liver inflammation and fibrosis, necessary features to inducing NAFLD/NASH progression. SCFA, as butyrate, acetate and propionate, are involved in the regulation of GLP-1 secretion by enteroendocrine L-cells. Butyrate is also an important microbiota metabolite that regulates insulin secretion from pancreatic β cells and modulates PPARγ signaling in the liver. The major component of incretin hormones family is GLP-1, which has a critical role in modulating hypoglycemia, insulin secretion, and reducing glucagon synthesis in pancreatic β cells; GLP-1 stimulates hepatic TLRs, promoting steatosis, inflammation fibrosis and IR. The figure was created using Biorender.com.

Although the GM metabolites are commonly recognized as “fuel” or energy sources in metabolic pathways, they also serve as intracellular signaling molecules, resembling neurotransmitters and hormones, that regulate enzyme activity [154]. Furthermore, many metabolites can inhibit enzymes in classical feedback loops of metabolic pathways or act as signaling molecules by binding to nuclear hormone receptors, such as PPARs and FXR [155]. The interaction between microbiota metabolites and the GLPA plays a crucial role in maintaining metabolic homeostasis in both states of health and disease. The main actors of the metabolic imbalance characterizing liver and pancreas diseases, as NAFLD, NASH, T2D and obesity are incretins. The European Association for the Study of the Liver’s (EASL) congress in Vienna recently announced that NAFLD disease will now be called metabolic dysfunction-associated steatotic liver disease (MASLD). The main reason is to better underline the multifactorial nature and the association of liver disease with obesity, T2D, chronic low inflammation, and the sustaining of gut dysbiosis and cardiometabolic risk factors [156]. Indeed, the main cause of MAFLD development is a prolonged imbalance of glucose, lipid, and cholesterol metabolism [157]. A growing number of in vitro, in vivo, and clinical studies suggest that MAFLD and MASH are strongly associated with the development of such metabolic diseases as obesity, T2D, dyslipidemias, and metabolic syndrome (SM) [158], and, reciprocally, patients with significant fibrosis in the context of MAFLD have been proven to be at risk of developing both T2D and arterial hypertension. Plasma BA concentrations were increased in MASH patients with severe IR [159] as well as obesity and T2D, where alterations in BA metabolism already occur. In the same way, other metabolites, as SCFAs, help to maintain an energy balance by regulating gut hormones such as gut trypsin peptide, GLP-1 and PYY, thereby preventing the development of metabolic diseases such as obesity, abnormal glucose and lipid metabolism, hypertension and NAFLD disease [160]. Overall, the interaction between incretins, the liver–pancreas axis, and the GM is complex and multifaceted. Certainly, responses to therapies utilizing SCFAs, BAs, and incretins can exhibit considerable variability among individuals. The treatments’ efficacy is contingent upon various factors, including the particular condition of the organ involved, the duration of therapy, and, most importantly, the unique characteristics of the patient. Thus, understanding how these components interact in health and disease may provide insights into the pathophysiology of metabolic disorders (Figure 3).

## 7. Microbiota-Derived Metabolites’ Involvement in Hepatic and Pancreatic Carcinomas Progression

Microbiota metabolites play significant roles in carcinogenesis, impacting a host’s risk in three main ways: (i) by modulating the equilibrium between host cell proliferation and death, (ii) shaping the functionality of the immune system, and (iii) affecting the metabolism of factors produced by the host. GM dysbiosis has been demonstrated to be crucial in the tumorigenesis of liver and pancreatic cancers. Indeed, the GM can influence cancer progression and could be considered a prognostic and predictive factor in the development of cancers, mainly HCC [161].

### 7.1. Microbiota and HCC

HCC is an aggressive malignancy and almost exclusively develops in patients with chronic liver disease and cirrhosis. As described above, the liver does not contain a microbiome, but it is closely connected to the gut via the portal venous system, constituting the gut–microbiota–liver axis [162]. Over the past decade, both experimental and clinical studies have elucidated the significant GM role in various stages of liver diseases, including the progression to liver cirrhosis and HCC. The majority of HCC cases arise in patients with pre-existing liver cirrhosis [163]. Pathological alterations occurring in liver cirrhosis, such as portal hypertension and reduced gastric acid secretion, directly compromise the intestinal barrier integrity and indirectly influence the GM composition. This disruption eases the translocation of pathogenic bacteria, contributing to the degeneration of liver diseases in HCC. The GM mechanisms favoring the hepatocarcinogenesis encompass factors like increased intestinal permeability (leaky gut), dysbiosis, activation of lipopolysaccharide (LPS)-TLR4 signaling, and alterations in bacterial metabolites [164]. In the liver, bacterial products stimulate TLRs, notably TLR-4, initiating the NF-kB pathway. This activation leads to a sustained initiation of mitogenic signaling and inhibition of programmed cell death. Prolonged exposure to various TLR ligands and other bacterial components due to chronic damage exposes the liver to inflammatory mediators, raising the progression of chronic liver disease and setting the stage for the eventual emergence of HCC [165]. Hepatocarcinogenesis in chronically injured livers is mainly dependent upon the GM and TLR4 activation in resident liver macrophages, although the promotion of early TLR4-dependent HCC is mainly mediated by the secretion of TLR4-dependent growth factors by HSCs [166]. As previously discussed, soluble fibers present in the diet undergo GM fermentation, resulting in the production of SCFAs, which are typically associated with health benefits. However, incorporating soluble fibers like inulin, as opposed to insoluble fibers, into a diet with a defined composition has been observed to induce HCC. Notably, this effect appears to be dependent on the GM composition, as evidenced by observations in multiple strains of dysbiotic mice. In fact, this phenomenon was not observed in germ-free mice or in mice treated with antibiotics, underscoring the crucial GM role in mediating the HCC development in response to dietary soluble fibers. Therefore, SCFAs and fermentable fibers did reduce adiposity and improve glycemic control, namely induction of cholestasis and HCC. Sodium butyrate (NaBu) was the first substance identified as enhancing histone acetylation. Numerous in vitro studies have indicated that NaBu possesses chemo preventive properties against various types of human cancer cells, including those of HCC [167]. Coradini et al. demonstrated that treatment of HCC-derived cells, specifically HepG2 and Hep3B, with butyrate for six days in vitro resulted in a substantial inhibition of proliferation. Accordingly, the authors observed that butyrate treatment effectively prevented the formation of liver metastasis in mice. This protective effect was linked to a preferential accumulation of butyrate in the liver and to its ability to induce histone acetylation [168,169]. The SCFA mechanism used to reduce cell proliferation by inhibiting histone deacetylases is now well established [170]. Nevertheless, NaBu is a histone deacetylase inhibitor (HDACi) with anti-cancer activity against HCC that is able to trigger cell cycle arrest, inhibits cell growth, and favors apoptosis [171]. NaBu may also reverse the malignant phenotype and prevent HCC cell migration and metastasis [172]. It was also shown that butyrate stimulated GLP-1 expression in human HCC cell line HepG2 [75]. In addition to butyrate, it has been verified that the other two SCFAs, acetate and propionate, also have a protective role against HCC. Via the gut–liver axis, acetate secreted by *Bifidobacterium pseudolongum* (*B. pseudolongum*) activates hepatic GPR43, which suppresses the oncogenic IL-6/JAK1/STAT3 signaling pathway and prevents disease progression [173]. In vitro, propionate and cisplatin co-treatment significantly induces apoptosis of HepG2 cells by increasing the autocrine expression of TNF-α via GPR41 signaling. From a clinical and translational perspective, a combination of propionate and cisplatin may have better therapeutic effects on HCC than conventional treatment, so the selective GPR41 agonist may be a candidate as an adjuvant therapeutic agent for HCC [174]. During the development of HCC, the accumulation of hydrophobic BAs, including CDCA, DCA and LCA, induce a variety of signals that improve hepatocyte death [175]. DCA results increased in the serum of patients with NASH and in animals with experimentally induced HCC. In vitro studies showed that DCA treatment induced cellular senescence in an HSC cell line (LX2) via induction of senescence-associated secretory phenotype (SASP) factors, reducing proliferation with cell cycle arrest at the G0/1 phase. In addition, treatment of the HCC cell line with the conditioned media of DCA-treated LX2 cells attenuated typical features of the tumor phenotype, such as cell migration and invasion [176]. Similarly, it has been demonstrated that UDCA/TGR5 binding can also suppress the progression of HCC [177].

Whole-body FXR-deficiency in mice promotes spontaneous hepatocarcinogenesis [178]. In FXR-null mice, intestinal selective reactivation of the FXR/FGF15 pathway restores BA homeostasis and inhibits spontaneous HCC development [179]. In instances of GM dysbiosis and inflammation, the intestinal FXR is downregulated, causing disruption in the enterohepatic circulation of BAs. Elevated levels of BAs within enterocytes can exacerbate intestinal inflammation. Additionally, hepatic inflammation triggers the activation of hepatic NF-κB signaling, which further suppresses FXR expression in the liver. Therefore, hepatic cholestasis and inflammation are aggravated, which might promote hepatocarcinogenesis [116]. TGR5, another important receptor for BAs mentioned above, is thought to play a role as well. The methylation of the TGR5 promoter is significantly frequent in HCC, and the hyper-methylated TGR5 serum cell-free DNA promoter may serve as a biomarker for HCC surveillance [180].

As previously mentioned, NAFLD can progress to HCC. In an HFD mouse model, Ex-4, an antidiabetic drug that targets GLP-1R, is protective against NAFLD. In this context, Ex-4 significantly improves obesity-induced hyperglycemia and hyperlipidemia and reduces HCC progression via suppression of proliferation and induction of apoptosis. Notably, Ex-4 stimulates protective functions through cAMP-PKA-EGFR-STAT3 signaling, resulting in suppression of genes, including c-Myc, cyclin D1, Bcl-2, and Bcl-xl. The same effects are also seen in vitro and in the HCC xenograft mouse model [181].

### 7.2. Microbiota and Pancreatic Cancers

Interestingly, a large number of epidemiological investigations confirmed that the occurrence of endometrial cancer, hepatobiliary cancer, pancreatic cancer, breast cancer, prostate cancer, and colorectal cancer are positively correlated with T2D [180,182].

In detail, the pancreatic ductal adenocarcinoma (PDAC) is the most prevalent neoplastic disease of the pancreas, accounting for more than 90% of all pancreatic malignancies [183]. Projections suggest a substantial increase in the incidence of PDAC in the future. One significant contributing factor to the projected increase in PDAC is the association with obesity and T2D. In recent studies, the evidence of bacterial and fungal populations in normal pancreatic tissue and PDAC samples were shown, and it was reported that the PDAC microbiome are different from healthy samples [184,185]. Ren et al. found that, in PDAC, there is a decrease in GM diversity. They observed changes in microbial profiling, particularly in abundant levels of *Prevotella*, *Veillonella*, *Klebsiella*, *Selenomonas*, *Hallella*, *Enterobacter*, *Cronobacter* and others, despite an increase in certain potential pathogens and bacteria-producing LPS [186]. Several studies have depicted that the microbiota diversity and alterations can be associated with PDAC development [187]. Inflammation can also contribute to PDAC development through its oncogenic effect. Chronic inflammation in pancreatic tissue can trigger a Kirsten rat sarcoma virus (KRAS) oncogenic mutation in insulin-positive endocrine cells and induce the differentiation of epithelial cells, resulting in PDAC [188]. The activated KRAS can further advance carcinogenesis by activating the NF-κB pathway [189]. Regarding the SCFA role, acetate and butyrate in particular can mitigate pancreatitis, offering protection against PDAC [190], which is associated with dysbiosis and characterized by a decrease in butyrate-producing bacteria [186]. Additionally, butyrate was found to have an anti-proliferative effect on cultured PDAC cells [191], and butyric acid can reduce the growth of cultured PDAC cells and activate differentiation [191]. Analogously, several in vitro studies have highlighted the potential therapeutic effects of butyrate and its analogs on PDAC cell lines. These studies have demonstrated that butyrate and its analogs exhibit pro-differentiating, anti-proliferative, pro-apoptotic, and anti-invasive effects. Additionally, butyrate acts as a histone deacetylases (HDACi), contributing to its anti-cancer properties, anti-inflammatory properties, and its anti-fibrogenic action [192,193]. On the other hand, acetic acid has a role in improving the invasiveness of PDAC cells by stimulating the epigenetic reprogramming of mesenchymal cells to cancer-related fibroblasts [191]. The dysregulation of SCFAs is strongly associated with PDAC and primarily involves the KRAS genes and the downstream pathways. In summary, butyrate influences the activation of NF-κB, which in turn downregulates p53 expression, influencing apoptosis and, therefore, PDAC progression [194]. SCFAs can activate FFA2 and FFA3 in intestinal epithelial cells, triggering the transmission of mitogen-activated protein kinase signaling, which in turn leads to the rapid secretion of chemokines and cytokines [195]. Additionally, GPR87, an LPA receptor, has been found to enhance PC aggressiveness by activating the NF-κB signaling pathway [196]. A recent mouse study documented a strong association between chronic pancreatitis and the GM [197]. This suggests that the interaction between the GM and BAs can significantly influence pancreatic tissue pathology and warrants further investigation. Therefore, research investigation into PC has demonstrated alternated BAs levels in patients [198,199]. Rees et al. compared the common bile duct BA composition in a PDAC and control group. The study reported that patients with PDAC tended to have elevated unconjugated BA levels [198], one of the main ways by which BAs induce PDAC progression is the upregulation of FXR [200]. Likewise, Lee JY et al. reported FXR to be highly expressed in five PC cell lines and PDAC specimens, suggesting its role in PC progression. The authors also reported a positive correlation of FXR expression with lymph node metastasis, cell proliferation, migration, and invasion [201]. Finally, the GM can modulate BA receptors through secondary BAs; for example, in PDAC cells, DCA induced STAT3 and epithelial growth factor receptor (EGFR) signaling by binding to TGR5 [202,203]. Therefore, targeting the BA receptors can be a potential intervention strategy.

The major role of GLP-1 in PC progression is decreasing blood glucose by promoting energy storage in adipose tissue and inhibiting glucagon secretion. Indeed, it has been demonstrated that the treatment with miRNAs released inhibiting PCSK1/3 expression, suppressing the GLP-1 production in enteroendocrine cells, which finally leads to increased IR [204,205]. Accordingly, hyperinsulinemia in diabetic patients seems to be the main reason for an increased risk of cancer [206]. The high levels of insulin have been shown to reduce the levels of the insulin growth factor (IGF-1) binding protein, which can tightly bind to IGFs, resulting in excessive free IGF-1 in cells and tissues. In turn, the IGF-1 levels have been shown to be associated with an increased risk of cancer [207]. PDAC has been observed to decrease insulin sensitivity in hepatic cells, resulting in reduced liver gluconeogenesis levels [208]. This manifests as a delayed and diminished insulin response in PDAC patients. Consequently, elevated blood glucose levels trigger increased insulin production and release from pancreatic β cells, causing endogenous hyperinsulinemia. Furthermore, due to the heightened IR, diabetic PDAC patients who rely on insulin therapy for blood sugar management experience exacerbated glucose control issues.

Koehler and Drucker explored the impact of GLP-1 activation on the growth and viability of human PDAC cells through both short-term in vitro studies and longer-term in vivo experiments on mice. They demonstrated that various human PDAC cell lines, including pancreatic ductal adenocarcinoma (CAPAN-1, CFPAC-1, and PL45) and carcinoma (Hs 7766T) cell lines, express GLP-1R. However, they observed distinctions in GLP-1R-mediated activation of ERK1/2 and cAMP, suggesting potential variations in the relative levels of GLP-1R expression on the surface of the different cell types under investigation [209]. As the ERK1/2 and, to a lesser extent, cAMP pathways are known to be involved in functions, including the regulation of meiosis and mitosis, and as they are also activated by carcinogens, the question raised is whether GLP-1RA might have pro-oncogenic characteristics.

**Figure 4:**
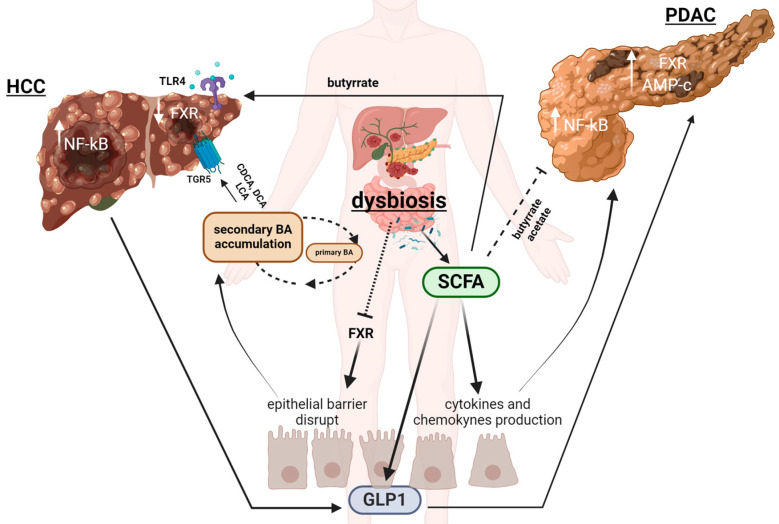
Microbiota metabolites trigger HCC and PDAC progression. The GM dysbiosis is one of the most important “hit” involves in neoplastic progression, especially in liver and pancreas. SCFA, BAs and GLP-1 GM metabolites play an important role in HCC and PDAC progression, mainly modulating the NF-kB and FXR hepatic and pancreatic pathways. In particular, SCFA increase NFkB pathways, binding FFA2 and TLR4 in the pancreas and liver, respectively. The GLP-1 incretin hormone, increased by both SCFA and indirectly by BAs, induces the activation of FXR and the downstream AMP-c e NFkB pathways in the liver and pancreas, respectively. GM metabolites are primarily able to increase cholestasis and inflammation in liver parenchyma, the secretion of cytokines and chemokines from intestinal lumen, and to induce a metastatic phenotype of PDAC cells. The figure was created using Biorender.com.

In addition, the liraglutide, a GLP-1 analog, was found to exhibit anti-tumor effects on the PANC-1 pancreatic cell line, reducing cell migration and invasion [210]. Liraglutide increased the chemosensitivity of PC cells to gemcitabine in the gemcitabine-resistant cell line PANC both in vitro and in vivo. In particular, treatment with liraglutide inhibited the proliferation and promoted apoptosis of cells in a dose-dependent manner and increased the expression of the GLP-1R and PKA [211]. Furthermore, in a PC xenograft model, Ex-4 treatment suppressed cell proliferation by attenuating pancreatic stellate cell function and suppressing extracellular matrix deposition [212]. In Figure 4, we summarize the different described pathologic crosstalk. Moreover, Table 1 highlights the GM metabolites (and associated bacteria) involved in NAFLD/NAH, T2DM, HCC and PDAC, which are the main pathways involved in their regulation.

**Table 1:** Summary of GM metabolites, microorganisms involved in NAFLD/NAH, T2DM, HCC and PDAC and main regulatory pathways involved.

	Metabolites	Microorganisms	Disease	Downstream Pathways
**SCFAs**	**acetate** **butyrate** **propionate**	Decreased by *Akkemansia muciniphila*, *Bacteroides* spp. *Bifidobacterium* spp., *Ruminococcus* spp., *Clostridium* cluster IV Increased by *Firmicutes (Eubacterium*, *Roseburia*, *Faecalibacterium*, *Ruminococcus* and *Caprococcus)*	**NAFLD/NASH** **T2D** **HCC** **PDAC**	--Activation of FFA2: increased hepatic lipogenesis and inhibits adypocite differentiation [74] --Activation of PPARα: prevents lipid accumulation [81,82] --Activation of FFA2: decrease in insulin secretion [20] --Downregulation of inflammatory cytokines (CCL-5 and TNFα) [128] --Improvment of insuline sensitivity [128] --Inhibition of histone deacetylase activity: inhibits cell growth and induces apoptotic cancer cell death [170,171,172] --Activation of GPR43: block of IL-6/STAT3/ signaling [173] --Inhibition of KRAS/NF-kB pathway: downregulation of P53 and stop PDAC progression [191,194]
**BAs**	**Primary BAs** (cholic acid and chenodeoxycholic acid) **Secondary BAs** (deoxycholic acid, lithocholic acid and ursodeoxycholic acid	BAs Deconjugation *Lactobacillus*, *Bacteroides*, *Clostridiumand listeria* BAs oxidation and epimeration *Eubacterium*, *Bacteroides*, *Escherichia*, *Egghertella* BAs esterification and desulfatation *Clostridium*, *Fusobacterium*, *peptococcus* and *Pseudomonas*	**NAFLD/NASH** **T2D** **HCC** **PDAC**	--FXR/TGR5 pathway: Maintains glucose homeostasis, reducing hepatic steatosis and promoting anti-inflammatory response [103] --PPAR signaling pathway, via FXR: Liver inflammation [117] --FXR/TGR5 signaling: intestinal environment stability and induces GLP1 secretion (glucose homeostasis) [143,149,150] --UDCA/TGR5 binding: Cell cycle and proliferation arrest and cell senescence [177] --FXR/FGF15 reactivation: Restores BA homeostasis and inhibited spontaneous HCC development [179] -Upregulation of FXR: lymph node metastasis, cell proliferation, migration, and invasion [200,201] --DCA-TGR5 binding: induced STAT3 and epithelial growth factor receptor (EGFR) signaling [202,203]
**GLP1**	**glucagon-like peptide-1 receptor 1**	Decreased by *Bacteroidetes* and *Firmicutes* Increased by *Bifidobacterium* spp., *Lactobacillus* spp. or *A. muciniphila*, *Bacteroides dorei*, *Lachnoclostridium* sp., *Mitsuokella multacida*	**NAFLD/NASH** **T2D** **HCC** **PDAC**	--increase in cAMP production: reduction in mRNA expression of stearoyl-CoA desaturase 1 and acetyl-CoA carboxylase [94,95] --stimulates glucose-dependent insulin secretion by β cells and downregulates secretion of glucagon by α cells: signaling pathways are both independent/dependent on PPARα [138] --Exendina-4 treatment: protective functions through cAMP-PKA-EGFR-STAT3 signaling [181] --GLP-1 analog, was found to exhibit anti-tumor effects: increased the expression of the GLP-1R and PKA [210,211]

## 8. GM manipulating Approaches

Since the relevant GM role in modulating the onset and progression of several metabolic disorders is linked to the liver–pancreas axis, in recent years, methods for shaping the GM composition have emerged. Among these, the fecal microbiota transplantation (FMT) and several dietary interventions are the most studied.

### 8.1. Fecal Microbiota Transplantation

Fecal microbiota transplantation, firstly described in the 4th century [213], involves transplanting fecal material from healthy donors to recipient patients, who need to re-establish a healthy composition and function of the GM [214]. At first, fecal microbiota was extracted from freshly collected feces and introduced directly into the gastrointestinal tract through endoscopic or non-endoscopic means [215]. Currently, FMT can be performed through various procedures, including colonoscopy, nasogastric or nasoenteric probe, capsules, or enemas, although there are contrasting points of view. Some favor oral administration, which is extremely simple, while others prefer to perform FMT via colonoscopy, as the feces are administered directly at the cecal level. The procedure choice may also be influenced by the patient, taking into account their preferences, available methods, age, and disease severity, as well as awareness of the risks associated with the procedure [216]. In recent years, different studies have shown that FMT is a successful therapy for recurrent and refractory *Clostridium Difficile* infection (rCDI) [217], in which the cure rate is up to 90% and correlates with an increase in overall survival and with a reduction in CDI-associated bloodstream infections and CDI-related surgery. For these reasons, in 2013 the U.S. Food and Drug Administration (FDA) approved the FMT for rCDI [218]. However, the use of FMT is actually much broader. In fact, FMT is currently being investigated as a potential treatment for other conditions associated with GM dysbiosis, such as inflammatory bowel disease (IBD) [216,219,220], neurodegenerative disorders [221], gynecological disorders [222], as well as metabolic disorders [223].

Although FMT is among the standard practice guidelines for rCDI treatment [224], it is not yet part of mainstream medicine. One potential explanation is the difficult process of donor selection. Initially, the ideal donor was thought to be a close member of the patient’s family, as it was assumed that they already shared several pathogens and some of the commensal organisms. To date, there is no evidence of this [225]. Moreover, locating a donor who satisfies rigorous health standards is challenging. Regarding exclusion criteria, they are further determined by the outcomes of screening and testing for additional systemic issues. These may encompass metabolic syndrome, diabetes, autoimmunity, irritable bowel syndrome, food intolerances, allergies, as well as neurological and psychiatric disorders, among others. Additionally, potential donors must not have a recent history of exposure to antibiotics [226].

To partially solve the problem of the donor shortage, in recent years the cryopreservation of fecal preparations has been implemented [227]. Due to the practice of freezing fecal samples, the shortage problem of material from donor patients could be avoided and safety precautions to mitigate the risk of transmitting multidrug-resistant bacteria can be heightened (e.g., quarantining fecal matter and expanding the screening of donors) [228]. The work protocol can also increase or decrease the FMT effectiveness [229]. Currently, there is a deficiency in uniform protocols for FMT, including stool processing methods and administration approaches. This standardization being missing may result in divergent treatment outcomes and impede comparisons across different studies. Typically, a stool sample collected from screened donors undergoes a thorough process at a stool bank, including preparation, division, and freezing into aliquots. The final fecal material is meticulously managed, labeled, tracked, and stored at −80 °C. On the FMT day, the frozen fecal suspension is thawed at +37 °C, mixed with normal saline to reach the desired volume, and the infusion should occur within 6 h of thawing [230]. Alternatively, freshly collected feces can be processed and used on the same day for the procedure, within 6 h of collection. According to protocols for the administration of fresh feces, they must be homogenized and diluted for easy administration, typically in sterile saline solution, but also in other diluents such as yogurt or milk [216].

Despite the now widely documented FMT effectiveness on different diseases, some doubts remain. Regarding its long-term safety, in 2019 two cases of invasive infections caused by multidrug-resistant bacteria and E. coli following FMT in immunocompromised patients were documented by the FDA [231]. Fortunately, adverse events after FMT are typically mild reactions such as diarrhea, abdominal pain, flatulence, and increased defecation, most of which resolve spontaneously. Due to the lack of a standardized FMT protocol, there is considerable variability in their clinical application. This can potentially lead to adverse reactions and ineffective treatment outcomes [232].

### 8.2. FMT and In Vivo Studies

The GM plays a crucial role in the gut–liver axis, and its alterations have been associated with several liver diseases [233,234,235,236,237]. Currently, there is no FDA-approved drug to treat NAFLD and NASH, the rates of which are increasing worldwide. It has been reported that patients with NAFLD and NASH show an altered microbiota, and dysbiosis can promote the NAFLD onset and its progression to cirrhosis and HCC [238,239].

In relation to this issue, several in vivo studies have suggested a benefit of FMT in liver disease. In a recent study, Zhou et al. have shown that FMT is able to attenuate NAFLD in mice fed with an HFD. In detail, they observed that HFD consumption significantly increases the proportions of CD3+CD4+ T cells, CD3+CD8+ T cells, and M1 macrophages in the liver, as well as the expression levels of other crucial enzymes involved in adipogenesis, thereby promoting the development of fatty liver. The FMT via oral gavage effectively decreased the levels of CD3+CD4+ T cells, CD3+CD8+ T cells, and M1 macrophages in the liver, ALT and AST levels, as well as histologically attenuating hepatic steatosis and lipid accumulations, thus improving NAFLD [240].

Another recent study showed that FMT improves NAFLD by restoring the GM. Fecal samples obtained from healthy donors were administrated to HFD-fed mice for 12 weeks. The FMT led to reductions in liver fat accumulation and body weight, and notably improved serum and liver biochemical indices in HFD-fed mice. Relative expression of liver mRNAs encoding inflammatory cytokines (IL-1β, IL-6, TNF-α, IFN-γ, and IL-1β) was significantly decreased following FMT compared to untreated HFD-fed mice. In addition, FMT improved the relative protein levels of intestinal barrier components, including claudin-1, occludin, and E-cadherin, and reduced serum LPS levels in mice. Furthermore, FMT reversed HFD-induced gut dysbiosis and increased the abundance of beneficial bacteria such as *Blautia* and *Akkermansia* [241]. Similarly, other studies highlight the positive FMT effect on mice with NAFLD, identifying that following treatment there is an improvement in the pathological condition in animals [242].

Mice transplanted with feces from NASH patients showed increased hepatic steatosis and inflammatory cell infiltration compared with those transplanted with feces from healthy human controls [243]. On the contrary, FMT has been shown to have a positive outcome. A study published in 2017 showed that FMT from lean mice to mice with NASH improved liver steatosis and inflammation in the recipient mice, suggesting a potential therapeutic FMT role. In detail, 200 μL of the supernatant from the fecal sample was administrated via gavage once daily for a duration of 8 weeks. After this time, NASH mice had a significant decrease in intrahepatic lipid accumulation and intrahepatic pro-inflammatory cytokines (IFN-γ and IL-17), while Foxp3, IL-4 and IL-22 were increased [244]. Likewise, in 2023 Lee et al. investigated how FMT mediates hepatic improvement in NASH. Mice fed with a high-fat, high-cholesterol and fructose (HFHCF) diet were transplanted via oral gavage with feces from pathogen-free mice every two days for 8 weeks. This intervention led to the suppression of hepatic pathogenic events, characterized by a reduction in inflammatory and fibrotic mediators. Additionally, FMT increased the expression of NF-E2-related factor 2 (NRF2), a key transcription factor that regulates antioxidant enzymes, in the livers of treated mice [245].

As is known, NAFLD can potentially advance to HCC. However, current treatment options for advanced HCC are scarce, emphasizing the need to explore innovative strategies. Recent animal models have further shown that the GM and its metabolites can directly impact both intrahepatic and peripheral inflammatory and immune responses in HCC [246,247].

The resistance to radiotherapy in HCC treatment had been suggested to be correlated with the disruption of the intestinal microbiome. Intestinal dysbiosis compromises anti-tumor immune responses by suppressing antigen presentation and inhibiting the functions of effector T cells through the cGAS-STING-IFN-I pathway [248]. Mice fed a high-fat, high-cholesterol (HFHC) diet for 14 months and who developed NAFLD-HCC exhibited GM dysbiosis, with distinct microbiota compositions being observed during the stages of disease progression. Specifically, there was a sequential increase in *Mucispirillum*, *Desulfovibrio*, *Anaerotruncus*, and *Desulfovibrionaceae*, while *Bifidobacterium* and *Bacteroides* were depleted in HFHC-fed mice, findings confirmed in human patients with hypercholesterolemia. Additionally, intestinal bacterial metabolites were found to be altered, including an increase in taurocholic acid and a decrease in 3-indolepropionic acid. Notably, atorvastatin administration, a hypoglycemic drug, restored cholesterol-induced intestinal microbiota dysbiosis and completely prevented the development of NAFLD-HCC [249]. Consequently, GM modulation is considered a potential method for preventing HCC [250].

The GM may also be involved in pancreatic diseases, both through direct bacterial colonization or through an indirect effect of molecules and toxins derived from microbial dysbiosis. Research suggests that alterations in the GM composition may contribute to the T2D development and its progression. In mouse models of T2D, FMT improved IR and inhibited apoptosis of pancreatic β cells [251]. In particular, T2D mice were orally administered 0.3 mL of fecal suspension per day for 8 weeks. The fecal samples were obtained from healthy mice and stored at −80 °C until the day of transplantation. After FMT, fasting insulin levels in T2D mice decreased and insulin resistance improved, as well as the insulin sensitivity index (HOMA-IS). The secretion of pro-inflammatory factors (IL-6 and TNF-α) decreased, while anti-inflammatory secretion increased in pancreatic tissues. Furthermore, the results of the HOMA-β index indicated that the size of the islets and the function of pancreatic β-cells were restored after FMT treatment in T2D mice [251]. Recently, Ding et al. also studied the FMT effect on T2D. After confirming through a prospective study that FMT from healthy donors improves glucose metabolism and insulin sensitivity in T2D patients [252], the authors assessed its therapeutic potential through in vivo studies. Genetically induced T2D mice were administered a fresh fecal suspension (0.2 mL/mouse) daily for 4 weeks, while the control T2D group received phosphate-buffered saline. The authors found that FMT improves clinical indicators of T2D, such as fasting plasma glucose, serum insulin, and oral glucose tolerance tests. Additionally, in T2D + PBS mice there was a decrease in *Ruminococaceae* and *Porphyromonadaceae* and an abundance of *Rikenellaceae* and *Lactobacillaceae*. FMT reversed this effect on the GM and improved intestinal barrier function, reduced inflammation, and led to alterations in the number of circulating immune cells [253].

In another study, Yang et al. induced T2D by feeding mice an HFD and administering low-dose streptozotocin injections over 4 weeks. The oral administration of a 0.3 mL bacterial solution had a therapeutic effect on T2D, improving both hyperlipidemia and hyperglycemia. Additionally, corticosterone, progesterone, L-urobilin, and other molecules were identified as biomarkers following FMT. Furthermore, bioinformatic analysis suggested that steroid hormone biosynthesis, arginine metabolism, proline metabolism, and unsaturated fatty acid biosynthesis could potentially regulate the effects of FMT [254]. From clinical evidence, it is hypothesized that GM modulating through FMT could potentially affect the systemic inflammation, immune responses, and metabolic pathways that may indirectly influence PDAC development and progression. Regarding this issue, it was demonstrated that FMT from mice with PDAC fast-tracks tumor progression in germ-free mice [184]. Riquelme et al. conducted a study of the tumor microbiome of PDAC patients and found that patients with long-term survival (LTS) had greater microbial diversity than those with short-term survival (STS). They identified three bacterial taxa enriched in patients with LTS, such as *Sachharopolyspora*, *Pseudoxanthomonas*, and *Streptomyces*. Furthermore, they found that FMT from LTS patients reduced tumor growth in mice compared with FMT from STS patients or healthy controls, suggesting that the microbiome of LTS patients may have a protective effect against tumors [255]. The study by Tintelnot et al. investigated chemotherapy response in PDAC patients, where less than half respond (R) while non-responders (NR) face severe consequences. They transplanted feces from R or NR patients into germ-free mice and found smaller tumors in mice with R patient microbiota post-chemotherapy. Analyzing intratumoral bacteria and serum, they discovered higher levels of the tryptophan metabolite 3-IAA in R patients and mice with R microbiota, suggesting its positive impact on chemotherapy outcomes [256]. Because of the presence of this solid gut–pancreas axis, these findings suggest that modulating the GM through FMT holds potential as a novel therapeutic strategy for pancreatic cancer.

### 8.3. FMT in Hepatic and Pancreatic Disease: Clinical Trials

Despite the high interest in the efficacy of FMT treatment for NAFLD, no significant clinical data have been reported to date. In a double-blind randomized controlled trial (RCT) by Witjes et al., 21 participants with hepatic steatosis underwent allogenic or autologous FMT via duodenal infusion three times over 24 weeks. Although there was a trend towards improved necro-inflammation scores, no significant enhancement in liver histology or biochemical parameters was observed post-allogenic FMT. However, significant alterations in the expression of hepatic genes linked to inflammation and lipid metabolism were noted in the allogenic FMT group compared to the autologous FMT group [257]. In another study by Xue et al., an open-label 4-week RCT involving 75 NAFLD patients was conducted. Participants were randomly assigned to receive allogenic FMT or oral probiotics, administered via colonic infusion for three days. While there were no significant differences in blood lipid levels or liver function tests pre- and post-treatment in either group, a notable decrease in liver fat attenuation degrees was observed in the FMT group compared to the non-FMT group. Microbiota analysis indicated a trend towards certain bacterial contents resembling those of healthy individuals after FMT. Moreover, FMT had a more pronounced impact on microbial community structure in lean NAFLD patients compared to obese NAFLD patients [258]. In addition to these promising results, ten clinical trials are currently underway to evaluate the effect of FMT treatment on NASH and NAFLD, and a study has been completed (Table 2). The study number NCT02496390 on NAFLD patients found that there were no significant changes in HOMA-IR or hepatic proton density fat fraction (PDFF) between those who received allogeneic FMT and those who received autologous FMT. However, those who received allogeneic FMT had a significant decrease in intestinal permeability six weeks after the procedure. Changes in fecal microbiota composition varied among individuals in both groups, but patients with improved intestinal permeability tended to have increased microbiota diversity. The study only administered FMT once and assessed efficacy after 24 weeks, making it difficult to predict long-term effects [259].

Regarding liver cirrhosis, liver transplantation remains the most effective treatment, boasting commendable survival rates. However, access to this treatment remains difficult for patients, often being limited to exceptional cases due to lengthy waiting lists. In this scenario, there is an urgent need to explore solutions for liver cirrhosis treatment [216]. FMT is increasingly emerging as a promising therapeutic approach for liver cirrhosis. Several studies have shown that intestinal microbiota transplantation can restore the GM balance, diminish inflammation, improve liver function, and enhance immune function [260]. The study number NCT03152188 involving 20 cirrhotic patients found that those who received FMT capsules from a donor enriched in *Lachnospiraceae* and *Ruminococcaceae* had improved outcomes compared to those who received placebo capsules after 5 months of treatment. The FMT group had reduced inflammation levels, with decreased serum IL-6 and LBP levels, improved cognitive scores, and changes in their gut microbiota that were correlated with cognitive improvement and decreased inflammation [261]. Thanks to these early results, FMT could be considered as a possible therapy for the treatment of liver cirrhosis. According to ClinicalTrials.gov, other clinical trials evaluating the efficacy and safety of FMT for NASH-related cirrhosis (NCT02868164 and CT02721264) and for cirrhosis are currently underway (Table 2).

Nowadays, therapies for advanced HCC are very limited, leading to the need to develop new therapies. As is known, the liver does not contain a microbiome. However, it is closely linked to the gut through the portal venous system, constituting the gut–microbiota–liver axis. It has been recently shown that, in HCC, there is an increase in the abundance of *Bacteroidetes*, *Lachnospiracea incertae sedis* and *Clostridium XIVa*, along with a reduction in *Verrucomicrobiaceae*, *Bifidobacteriaceae*, *Akkermansia*, and *Bifidobacterium*, as well as enhanced intestinal inflammation. Therefore, there was a weaker anti-tumor inflammatory response of the host liver [262,263]. Following these findings, several studies have evaluated the potential predictive and prognostic role of GM composition in HCC [264,265]. In vivo studies have shown that the GM and its metabolites have a direct impact on both intrahepatic and peripheral inflammatory and immune responses in HCC. This influence can lead to changes clinical course and overall survival, mainly by affecting the functions of effector and regulatory T cells [266]. Therefore, GM modulation can be considered as an approach to prevent HCC. Concerning the interaction between immune response and GM, two clinical trials aimed at evaluating FMT in patients with HCC are ongoing. In detail, the main purpose of these studies is to test the safety and efficacy of FMT combined with standard immunotherapy, even in patients who had not responded to previous immunotherapy for advanced HCC (NCT05750030 and NCT05690048) (Table 2).

Based on the current literature, it is now well known that dysbiosis is a risk factor for T2D. This association has thus opened a new perspective for potential new therapies for T2D, and recent studies have shown that FMT improved the metabolic profiles of T2D. Comparing FMT alone and dual treatment FMT + metformin in patients with T2D, both treatments were found to significantly improve clinical indicators of HOMA-IR and body mass index (BMI), fasting and postprandial blood glucose, and hemoglobin A1c (HbA1c). FMT with or without metformin also significantly improves the IR, BMI, and GM communities of patients with T2D through colonization of donor-derived microbiota [267]. Repeated FMT improves the level and duration of microbiota engraftment in obese T2D patients. Combining the lifestyle intervention with FMT led to more favorable changes in recipients’ GM and improved lipid profile and liver stiffness [268]. Furthermore, the comparison of diet and dietary treatment combined with FMT showed that both treatments have great potential in controlling blood glucose and blood pressure levels. FMT changed the GM more rapidly than diet: beneficial bacteria, such as *Bifidobacterium*, increased, and were negatively correlated with blood glucose, blood pressure, blood lipids and BMI; in contrast, sulfate-reducing bacteria (SRB), *Bilophila* and *Desulfovibrio*, which decreased significantly after treatment, showed a positive correlation with blood glucose indices. Therefore, personalized diet is useful for improving blood glucose control in T2D patients and has also shown the potential to reverse dyslipidemia and dysarteriotonia [269]. Additionally, four studies regarding T2D associated with FMT therapy are underway (Table 2), and one has been completed (NCT03127696). This study, conducted on 61 patients, found that FMT combined with lifestyle intervention (LSI) resulted in the highest percentage of patients acquiring lean donor microbiota. Additionally, repeated FMT significantly increased the establishment of lean-associated microbiota (*p* < 0.05). FMT, with or without LSI, augmented butyrate-producing bacteria, while the combination of LSI and FMT led to an increase in *Bifidobacterium* and *Lactobacillus* (*p* < 0.05). The combination of FMT + LSI also resulted in reduced cholesterol levels and liver stiffness compared to baseline (*p* < 0.05) [268].

A specific intestinal and tumor microbiome has been observed in mouse models of PDAC, which can be remodeled using antibiotics capable of inducing T cell activation, improving immune surveillance and increasing sensitivity to immunotherapy. The presence of *Gammaproteobacteria*, a bacterium capable of metabolizing gemcitabine in its inactive form, was demonstrated in PDAC, suggesting that its presence in PDAC could cause tumor resistance to this therapy. Similarly, most chemotherapeutic and immunotherapeutic agents, effective in other malignancies, have limited efficacy in PDAC treatment [255]. Several pieces of evidence suggest that GM modulation leads to greater efficacy of anti-tumor therapy [256]. Accordingly, the modulation of the gut and/or tumor microbiome could emerge as a new strategy in PDAC. Regarding this issue, one study is ongoing to evaluate the safety, tolerability, and feasibility of FMT in resectable patients with PDAC (NCT04975217) (Table 2). Specifically, patients will undergo FMT via a colposcopy and receive FMT capsules orally once a week for 4 weeks in the absence of disease progression or unacceptable toxicity. Patients will then undergo standard tumor resection and will be followed up at 2 weeks and 30, 60, 90 and 180 days after surgery. The results seem promising. The importance of FMT in pancreatic disorders is summarized in this interesting review [270].

**Table 2:** Clinical trial reports regarding FMT treatment of liver and pancreatic diseases reported on https://clinicaltrials.gov (accessed on 20 September 2023).

Disease	Number	Title	Status
NAFLD	NCT05607745	Dietary Counseling Coupled With FMT in the Treatment of Obesity and NAFLD—the DIFTOB Study	ACTIVE, NOT RECRUITING
NAFLD	NCT04594954	Effects of Fecal Microbiota Transplantation on Weight in Obese Patients With Non-alcoholic Fatty Liver Disease	UNKNOWN
NAFLD	NCT06024681	Impact of FMT on the Phenome in Patients With NAFLD and Fibrosis	RECRUITING
NAFLD	NCT02496390 [259]	Transplantation of Microbes for Treatment of Metabolic Syndrome and NAFLD	COMPLETED
NAFLD	NCT03648086	Intestinal Microbiota Transplantation for Non-alcoholic Fatty Liver Disease	UNKNOWN
NAFLD	NCT04465032	The Effect of Consecutive Fecal Microbiota Transplantation on Non-Alcoholic Fatty Liver Disease (NAFLD)	UNKNOWN
NASH	NCT02469272	Fecal Microbiota Transplantation (FMT) in Non-alcoholic Steatohepatitis(NASH). A pilot study	UNKNOWN
NASH	NCT05821010	Synbiotics and Fecal Microbiota Transplantation to Treat Non-Alcoholic Steatohepatitis	RECRUITING
NASH	NCT03803540	Fecal Microbiota Transplantation for the Treatment of Non-Alcoholic Steatohepatitis	NOT YET RECRUITING
NASH	NCT05622526	Evaluate the Efficacy, Safety and Tolerability of Fecal Microbiota Transfer for the Treatment of Patients With Non-alcoholic Steatohepatitis	NOT YET RECRUITING
NASH and Cirrhosis	NCT02868164	Fecal Microbiota Therapy Versus Standard Therapy in Decompensated NASH-Related Cirrhosis: A Randomized Controlled Trial	WITHDRAWN
NASH and Cirrhosis	NCT02721264	Fecal Microbiota Therapy Versus Standard Therapy in NASH-Related Cirrhosis.	UNKNOWN
Cirrhosis	NCT03796598	FMT in Cirrhosis and Hepatic Encephalopathy	COMPLETED
Cirrhosis	NCT03152188 [261]	Oral Fecal Transplant in Cirrhosis	COMPLETED
Cirrhosis	NCT04932577	Fecal Microbiota Transplantation for Liver Cirrhosis	RECRUITING
Cirrhosis	NCT02862249	Trial of Fecal Microbiota Transplantation in Cirrhosis	UNKNOWN
Cirrhosis	NCT03014505	Fecal Microbiota Transplantation for Decompensated Cirrhosis	UNKNOWN
Cirrhosis	NCT04842539	Fecal Microbiota Transplantation in Decompensated Cirrhosis	COMPLETED
HCC	NCT05750030	FMT in IT-refractory HCC—FAB-HCC Pilot Study	RECRUITING
HCC	NCT05690048	Fecal Microbiota Transfer in Liver Cancer to Overcome Resistance to Atezolizumab/Bevacizumab (FLORA)	NOT YET RECRUITING
T2D	NCT03127696 [268]	Randomized Placebo-controlled Study of FMT to Impact Body Weight and Glycemic Control in Obese Subjects With T2D	COMPLETED
T2D	NCT05253768	Safety and Efficacy of Human Microbiota Transplantation for Overweight and Obese Type 2 Diabetes Mellitus	UNKNOWN
T2D	NCT02346669	Fecal Microbiota Transplantation for Diabetes Mellitus Type II in Obese Patients	UNKNOWN
T2D	NCT01790711	Fecal Microbiota Transplantation on Type 2 Diabetes Mellitus	UNKNOWN
T2D	NCT06192693	Fecal Microbiota Transfer to Improve Diabetes Control Post-bariatric Surgery	NOT YET RECRUITING
PC	NCT04975217	Fecal Microbial Transplants for the Treatment of Pancreatic Cancer	RECRUITING
PC	NCT05606523	Microbiota and Pancreatic Cancer Cachexia	RECRUITING

### 8.4. Additional Treatments to Modulate the GM in Liver and Pancreatic Disease

It has been widely demonstrated that diet has a significant impact on the composition and stability of the GM, and different diets affect the species of microorganisms that populate the gut differently, changing its internal balance [271]. Consequently, there is a change in the level of metabolites derived from the microorganisms present, among them SCFAs, incretins and BAs. In mice fed with a HFHC diet, high dietary cholesterol was shown to lead to sequential progression of steatosis, steatoepatitis, fibrosis, and HCC simultaneously with IR, all associated with GM dysbiosis. In particular, dietary cholesterol induced altered intestinal bacterial metabolites, including increased taurocholic acid and decreased 3-indolepropionic acid [249]. In a mouse model of acute necrotizing pancreatitis, a Western diet feeding intensified the pathology by increasing bacterial spread and altering the intestinal metabolic profile with SCFAs, whereas butyrate supplementation attenuated disease progression [272]. Thus, while a HFD can cause different kinds of pathological changes, a healthy diet rich in fiber (fruits, vegetables, whole grains, and legumes, which belong to the category of prebiotics) promotes the growth of beneficial bacteria [273,274].

In addition to diet, another therapeutic option to restore the GM equilibrium is the use of probiotics [275], live beneficial bacteria, that can perform a wide variety of functions, including antioxidant, anti-tumor, anti-inflammatory elements and improving metabolism and immunological function [276,277]. These properties may be relevant to liver health, especially in conditions characterized by inflammation. Several research reports suggest that probiotics may have a preventive role in liver diseases, including conditions that can progress to liver cancer, such as NAFLD. Wang et al. have shown that supplementation of *Bifidobacterium bifidum V* (*BbV*) and *Lactobacillus plantarum X* (*LpX*) in mice with NAFLD significantly reduced hepatic levels of total cholesterol and total TGs [278]. Similarly, the administration of *Bacteroides thetaiotaomicron* (*B. theta*) reduced body weight, fat accumulation, hyperlipidemia and IR, and it also prevented hepatic steatohepatitis and liver damage. *B. theta* also increased liver and intestinal folate levels, hepatic metabolites, and the percentage of polyunsaturated fatty acids in the liver, offering a widely reported benefit for improving NAFLD [279]. In mice with NAFLD-HCC, the supplementation of *Lactobacillus acidophilus*, through the action of the metabolite valeric acid, which binds to the hepatocyte surface GPR41/43 receptor to inhibit the Rho-GTPase pathway, inhibited NAFLD-HCC [280]. Likewise, the acetate secreted by *Bifidobacterium pseudolongum*, and reaching the liver via the portal vein, has a protective role in NAFLD-HCC progression and can therefore be thought of as a potential new probiotic [173]. The pancreas, an organ involved in both digestive and endocrine functions, can also be influenced by the GM through the gut–pancreas axis. Although gut dysbiosis is increasingly recognized as a pathophysiological component of metabolic syndrome, the role and mode of action of specific gut microbes remain unknown. Rampanelli et al. have previously shown that the commensal butyrogenic *Anaerobutyricum soehngenii* (*A. soehngenii*) is associated with improved insulin sensitivity in subjects with metabolic syndrome, and in a subsequent clinical trial they investigated its potential therapeutic effects in patients. The treatment with *A. soehngenii* caused a marked increase in the postprandial excursion of GLP-1 and an increase in plasma secondary BAs, which were positively associated with GLP-1 levels, improving glycemic control within 24 h [281].

In recent years, several studies have shown that dysbiosis and gut inflammation are very common in T2D patients [282]. In this context, probiotics and prebiotics, used as dietary supplements, can help to improve these conditions by increasing the production of hypoglycemic hormones, such as incretin, and reducing IR and blood glucose levels [283]. In several human and animal studies, it has been seen that specific gut bacteria and their metabolites are significantly altered in T2D. A decrease in butyrate-producing microorganisms and an activation of the LPS component, causing endotoxemia, have been described in T2D patients [284]. Microbial metabolites, SCFA, and total BAs can activate hypoglycemic signaling pathways, improve endotoxemia, and increase GLP-1 and PYY levels. Some antidiabetic drugs, such as metformin, can improve metabolic dysfunction by activating the GUDCA-intestinal FXR pathway through *Bacteroides fragilis* [285]. In db/db mouse models, the antidiabetic effect of 14 probiotics was demonstrated through significant improvement in blood glucose and blood lipid parameters, as well as morphological changes in the pancreas and liver. Probiotics increased the levels of SCFA-producing bacteria and decreased the levels of Escherichia coli and LPS. They also enhanced insulin secretion through glucose-triggered GLP-1 secretion and protected the pancreas from apoptosis, probably through upregulation of the PI3K/AKT pathway [286,287]. Severe AP is a critical disease characterized by a severe systemic inflammatory response resulting in persistent multiorgan failure and sepsis. Li et al. demonstrated that bifidobacteria, particularly *Bifidobacterium animalis* (*B. animalis*) and its metabolite lactate, protect against AP by regulating pancreatic and systemic inflammation in different mouse models. In support of these findings, AP patients showed reduced fecal abundance of bifidobacteria, which was inversely correlated with the severity of systemic inflammatory responses. These findings suggest that *Bifidobacterium* spp. and their metabolite lactate protect against acute pancreatitis via inhibition of pancreatic and systemic inflammatory responses [288]. As previously reported, the PC patients with PC gut dysbiosis and gut mucosal barrier dysfunction. Recently, it was found that intestinal care using L-glutamine-enriched oral supplements and probiotics, including *Lactobacillus casei supplement Shirota* (*Yakult*^®^) and *Clostridium butyricum strain MIYAIRI 588* (*Miya*-*BM*^®^), could induce a strong anti-tumor immune response through induction of fully mature tertiary lymphoid structures in some PC patients who received three cycles of preoperative chemotherapy [289]. In addition to prebiotics and probiotics, there are symbiotics and postbiotics. Symbiotics are given by a combination of probiotics and prebiotics, thus live microorganisms and substrates selectively used by host microorganisms. This dual approach aims to provide a favorable environment for the growth and maintenance of the beneficial bacteria introduced through probiotic supplementation. Clinical studies have shown that symbiotic supplementation improved steatosis in NAFLD patients [290] and also reduced blood glucose levels and improved IR in patients with prediabetes and T2D [291]. In addition, in T2D patients, the symbiotic supplementation improved the gut microenvironment and may be a potential adjunctive therapy to improve metabolism and lower HbA1c [292]. Postbiotics are compounds of inanimate microorganisms and/or their cellular components with or without metabolites. Postbiotics have the potential to modulate gut barrier stability and ameliorate metabolic diseases. In mouse models, simultaneous use of *Lactobacillus paracasei*-derived postbiotics and HFDs was shown to slow weight gain, suppress IR, and improve indicators of blood lipid metabolism and liver steatosis, suggesting a protective role against NAFLD [293]. Finally, the use of two *Lactobacillus brevis strains* (*KLDS 1.0727* and *KLDS 1.0373*) capable of synthesizing the postbiotic gamma-aminobutyric acid (GABA) was seen to reduce hyperglycemia and hyperlipidemia in a mouse model of diabetes [294].

## 9. MicroRNA

MicroRNA (miRNA), small non-coding RNA molecules of about 21–25 nucleotides, play a crucial role in the post-translational regulation of gene expression in various biological contexts, such as development, differentiation, proliferation, apoptosis, and metabolism. Consequently, the dysregulation of miRNA expression or function is associated with different diseases, including cancer and cardiovascular/neurological/metabolic disorders [295,296,297]. For this reason, in last years they have emerged as potential therapeutic targets and diagnostic biomarkers in various diseases [298].

Several studies suggest that GM-miRNA interactions may play a critical role in liver dysfunction and disease. It is known that the severity of liver disease is characterized by the expression of specific miRNAs and that the GM is a key factor in both liver disease and miRNA expression [299]. In detail, it has been shown that miRNA-21 promoted liver dysfunction by affecting intestinal lactobacilli in mice, whereas mice lacking miRNA-21 had reduced liver damage and were protected from small intestinal injury and GM dysbiosis [300]. In addition, miRNA and GM are likely to play relevant roles in liver diseases, in the forms of NAFLD and liver fibrosis, through complex interactions and modulations of various biological processes such as inflammation, lipid metabolism, and cell proliferation [301]. The miR-21 role on lipid metabolism and chronic liver diseases has been well documented [302]. On the other hand, miR-21 deficiency has been shown to have a hepatic protective effect through GM modulation, regulating intestinal epithelial cell tight junctions and reducing endotoxemia [300]. Moreover, increasing evidence suggests that miRNAs, through interactions with the GM, play a key role in HCC development [303].

A study showed the close link between GM and HCC [304]. Specifically, in mice treated with a streptozotocin-rich fat diet (STZ-HFD), it was observed that the sex-based disparity in liver carcinogenesis was associated with the GM, BAs, and tumor-suppressing miRNAs (miR-26a, miR-26a-1, miR-192, miR-122, miR-22, and miR-125b). In male mice, the microbiota regulated BAs and miRNAs that promote HCC via an unclear regulatory mechanism. On the other hand, in female mice, high levels of FXR, a nuclear receptor for BAs, may increase the expression of tumor suppressor miRNAs such as miR-26a, miR-26a-1 and miR-122, possibly decreasing the risk of HCC developing [304]. Furthermore, butyrate has been shown to induce cell apoptosis via upregulation of miR-22 and repression of sirtuin1 expression in HCC liver cells [305]. However, some probiotics have the ability to negatively regulate the expression of oncogenic miRNAs in mice with HCC tumors [306]. Recent research has focused on the implication of miRNAs in crosstalk between the GM and pancreatic function. Haniff et al. designed small molecules that can selectively block a specific member of the miRNA-200 family implicated in the pathology of T2D. Aiming at the pre-structuremir-200c, the small molecule can improve insulin sensitivity and blood sugar levels by reversing the pro-apoptotic effects in β pancreatic cells in a mouse model. This suggests that miR-200c plays a crucial role in T2D, making it a potential target for therapies for diabetes [307]. In addition, it was found that miR-155 was upregulated in mice with severe PA, in association with an increase in tissue inflammatory factors, a decrease in intestinal barrier proteins, and aggravated intestinal injury. Through GM regulation and activation of the TLR4/MYD88 pathway, miR-155 lead to the release of inflammatory mediators and regulated pancreatic damage. However, inhibiting miRNA-155 in mice resulted in a reduction in pancreatic injury and inflammation, resulting in improved histopathological damage [308]. Different studies suggest that miRNAs may play a role in the development of pancreatic tumors by affecting inflammation, immune response, and tumor growth. MicroRNA have a significant impact on both the progression and repression of PDAC through various signaling pathways, such as proliferation, cell differentiation, cell apoptosis, migration, and metastasis creation [309]. Indeed, several miRNAs have been found to be upregulated in pancreatic cancer, including miR-21, miR-155, and miR-196a, and others have been found to be downregulated, such as miR-34a [310]. Wang et al. discovered that miRNAs and certain GM bacteria can interact with each other, potentially contributing to the development of pancreatic cancer [311]. The combined use of specific bacteria and miRNAs may offer a way to inhibit the progression of PDAC [311]. Accordingly, Shirazi et al. demonstrated that *H. pylori* and *P. gingivalis* have a relevant impact on the cell cycle, DNA damage, miRNA expression, and epigenetics in pancreatic cancer [312]. In conclusion, the GM and miRNAs can be involved in the development of pancreatic disease, and future studies are needed to characterize these interactions and identify potential therapeutic pathways.

## 10. Discussion

In the last ten years, too many types of approaches have been proposed to regulate dysbiosis GM in order to ameliorate and prevent liver and pancreatic metabolic disorders. The main molecules proposed in the treatment of NAFLD and T2DM are the GLP-1R and FXR agonists because of their ability to regulate numerous metabolic activities in both the liver and pancreas. Indeed, GLP-1RA have shown promise in ameliorating hepatic insulin resistance and improving NAFLD by positively influencing hepatic lipid metabolism. These agents, currently approved for managing diabetic hyperglycemia and obesity, have been observed to provide beneficial effects on liver enzymes, promote weight loss, and enhance hepatic metabolism. Given these properties, GLP-1RAs might serve as effective treatments for NAFLD and NASH in both diabetic and nondiabetic individuals. In addition, FXR agonists, including synthetic bile acids and steroidal and non-steroidal compounds, have gained interest as potential therapeutic agents for various metabolic disorders, including NAFLD, NASH and T2DM. By targeting FXR, these agonists aim to modulate bile acid homeostasis and lipid metabolism, potentially offering benefits in managing these conditions. Another therapeutic way for restoring GM eubiosis, aside from dietary adjustments, involves probiotics administration. Indeed, probiotics and prebiotics, used as dietary supplements, can help to improve these pathological conditions by increasing the production of hypoglycemic hormones such as incretin, and reducing IR and blood glucose levels.

The current literature suggests that FMT could improve metabolic states and, in particular, insulin sensitivity, yet the impact of FMT on weight loss in obese subjects needs further study. In addition, FMT is also being studied as a potential treatment for other conditions associated with intestinal dysbiosis, such as inflammatory bowel diseases, neurodegenerative disorders, gynecological disorders and metabolic disorders, but not only. Due the importance of GM metabolites in maintaining healthy homeostasis, intestinal dysbiosis is strongly implicated in the disease scenario. This is because GM and its metabolites can affect inflammatory and immunity in different contexts. At the tumor level, in both HCC and PDAC, intestinal dysbiosis affects treatment outcomes and survival, and several studies have shown the FMT therapeutic potential in terms of both disease progression and response to therapy [250,255].

Overall, FMT could therefore be considered as a new therapeutic approach for liver and pancreatic diseases, although further studies are definitely needed to assess their effectiveness and safety in treating these conditions. Despite all this interest in FMT therapy, there are still some key points that need to be improved, such as procedural and long-term safety, potential pathogen transmission, and standardization of workflow [313]. In order to improve the FMT safety and effectiveness, new protocols have recently been introduced. Washed microbiota transplantation (WMT) and filtered fecal transplantation (FFT) represent two new transplantation methods involving washing and filtration steps and, consequently, a lower risk of contamination and transmission of pathogens [314]. In addition, the concept of “bacterial consortium” has recently been coined, already emerging as a potentially effective approach in treating CDI infection. The implementation of specific interventions based on individual health conditions, microbial profiles and responses to specific dietary and lifestyle changes could offer a more personalized approach and therefore be more functional. Furthermore, not only FMT, but also a healthy diet rich in fiber and the integration of prebiotics, probiotics, symbiotics and postbiotics promote antioxidant, anti-inflammatory, anti-tumor behaviors, improving the response of the immune system in different diseases of the liver and pancreas [315,316].

In this complex scenario, the interaction between miRNAs and the specific bacteria in the GM may also contribute to regulating the GLPA; therefore, characterizing the interactions between the GM and miRNAs is essential in identifying potential therapeutic targets for liver and pancreatic diseases.

Taken as a whole, these results make us understand how the GM diversity can affect human health. It is therefore critical to study it more in depth in order to build more and more personalized and effective therapeutic strategies for each patient.

## 11. Conclusions

The gut–liver–pancreas axis (GLPA) is a complex network of organs finely regulated by various factors and hormones. In this scenario, a critical role is played by the GM, which produces, transforms and regulates the various molecules responsible for maintaining the systemic metabolic host state. In particular, SCFA, BA, and GLP-1 are able to trigger physiological and pathological functions in the host, thus affecting the GLPA.

Due to the intricate functions of the many molecules involved in regulating the GLPA in states of health and disease, addressing these pathologies requires a comprehensive approach. The use of innovative approaches such as FMT and other microbiota modulation strategies that influence hormone and metabolite levels highlight the need to advance personalized medicine. We believe that this evolution is essential to improving therapeutic outcomes in the management of metabolic diseases.

## 12. Limits of the Study

Understanding the role of the microbiota in the gut–liver–pancreas axis in health and disease is a complex challenge. This is due to the large number of specific microorganisms and metabolites present in the GM, each of which can perform a multitude of functions within the body. Consequently, significant time and numerous studies, both basic and clinical, will be needed to fully understand their role in the various pathways in which they are involved. Another complicating factor is the individuality of the GM. It is well established that an individual’s GM differs from that of another individual and that, over the course of a lifetime, an individual’s microbiota may also change, as it depends on age, diet, lifestyle, and more. Therefore, the different therapeutic approaches used to restore the proper structure of the GM must vary from individual to individual and cannot be considered “universal”. Going forward, it is therefore important to remember that therapies must be individualized. Although the study of GM in liver and pancreatic health and disease is becoming increasingly important in the field of research, in order to better understand this topic and identify potential solutions, it is absolutely necessary to develop innovative methods to study the individual’s microbiota over time and to better understand the interactions it may have with the various diseases present.

## Data Availability

Data sharing is not applicable to this article.

## References

[1] Hooper L.V., Gordon J.I. (2001). Commensal host-bacterial relationships in the gut. Science.

[2] Johnson K.V. (2020). Gut microbiome composition and diversity are related to human personality traits. Hum. Microb. J..

[3] Ding T., Schloss P.D. (2014). Dynamics and associations of microbial community types across the human body. Nature.

[4] Thomas S., Izard J., Walsh E., Batich K., Chongsathidkiet P., Clarke G., Sela D.A., Muller A.J., Mullin J.M., Albert K. (2017). The Host Microbiome Regulates and Maintains Human Health: A Primer and Perspective for Non-Microbiologists. Cancer Res..

[5] Arpaia N., Campbell C., Fan X., Dikiy S., van der Veeken J., deRoos P., Liu H., Cross J.R., Pfeffer K., Coffer P.J. (2013). Metabolites produced by commensal bacteria promote peripheral regulatory T-cell generation. Nature.

[6] Louis P., Hold G.L., Flint H.J. (2014). The gut microbiota, bacterial metabolites and colorectal cancer. Nat. Rev. Microbiol..

[7] Reichardt N., Duncan S.H., Young P., Belenguer A., McWilliam Leitch C., Scott K.P., Flint H.J., Louis P. (2014). Phylogenetic distribution of three pathways for propionate production within the human gut microbiota. ISME J..

[8] Morrison D.J., Preston T. (2016). Formation of short chain fatty acids by the gut microbiota and their impact on human metabolism. Gut Microbes.

[9] Layden B.T., Angueira A.R., Brodsky M., Durai V., Lowe W.L. (2013). Short chain fatty acids and their receptors: New metabolic targets. Transl. Res..

[10] Kimura I., Ichimura A., Ohue-Kitano R., Igarashi M. (2020). Free Fatty Acid Receptors in Health and Disease. Physiol. Rev..

[11] Cani P.D., Dewever C., Delzenne N.M. (2004). Inulin-type fructans modulate gastrointestinal peptides involved in appetite regulation (glucagon-like peptide-1 and ghrelin) in rats. Br. J. Nutr..

[12] Lin H.V., Frassetto A., Kowalik E.J., Nawrocki A.R., Lu M.M., Kosinski J.R., Hubert J.A., Szeto D., Yao X., Forrest G. (2012). Butyrate and propionate protect against diet-induced obesity and regulate gut hormones via free fatty acid receptor 3-independent mechanisms. PLoS ONE.

[13] Gao Z., Yin J., Zhang J., Ward R.E., Martin R.J., Lefevre M., Cefalu W.T., Ye J. (2009). Butyrate improves insulin sensitivity and increases energy expenditure in mice. Diabetes.

[14] Frost G., Sleeth M.L., Sahuri-Arisoylu M., Lizarbe B., Cerdan S., Brody L., Anastasovska J., Ghourab S., Hankir M., Zhang S. (2014). The short-chain fatty acid acetate reduces appetite via a central homeostatic mechanism. Nat. Commun..

[15] Tolhurst G., Heffron H., Lam Y.S., Parker H.E., Habib A.M., Diakogiannaki E., Cameron J., Grosse J., Reimann F., Gribble F.M. (2012). Short-chain fatty acids stimulate glucagon-like peptide-1 secretion via the G-protein-coupled receptor FFAR2. Diabetes.

[16] Anastasovska J., Arora T., Sanchez Canon G.J., Parkinson J.R., Touhy K., Gibson G.R., Nadkarni N.A., So P.W., Goldstone A.P., Thomas E.L. (2012). Fermentable carbohydrate alters hypothalamic neuronal activity and protects against the obesogenic environment. Obesity.

[17] Chambers E.S., Viardot A., Psichas A., Morrison D.J., Murphy K.G., Zac-Varghese S.E., MacDougall K., Preston T., Tedford C., Finlayson G.S. (2015). Effects of targeted delivery of propionate to the human colon on appetite regulation, body weight maintenance and adiposity in overweight adults. Gut.

[18] Nøhr M.K., Pedersen M.H., Gille A., Egerod K.L., Engelstoft M.S., Husted A.S., Sichlau R.M., Grunddal K.V., Poulsen S.S., Han S. (2013). GPR41/FFAR3 and GPR43/FFAR2 as cosensors for short-chain fatty acids in enteroendocrine cells vs FFAR3 in enteric neurons and FFAR2 in enteric leukocytes. Endocrinology.

[19] Bolognini D., Moss C.E., Nilsson K., Petersson A.U., Donnelly I., Sergeev E., König G.M., Kostenis E., Kurowska-Stolarska M., Miller A. (2016). A Novel Allosteric Activator of Free Fatty Acid 2 Receptor Displays Unique Gi-functional Bias. J. Biol. Chem..

[20] Kimura I., Miyamoto J., Ohue-Kitano R., Watanabe K., Yamada T., Onuki M., Aoki R., Isobe Y., Kashihara D., Inoue D. (2020). Maternal gut microbiota in pregnancy influences offspring metabolic phenotype in mice. Science.

[21] Priyadarshini M., Villa S.R., Fuller M., Wicksteed B., Mackay C.R., Alquier T., Poitout V., Mancebo H., Mirmira R.G., Gilchrist A. (2015). An Acetate-Specific GPCR, FFAR2, Regulates Insulin Secretion. Mol. Endocrinol..

[22] Cani P.D., Amar J., Iglesias M.A., Poggi M., Knauf C., Bastelica D., Neyrinck A.M., Fava F., Tuohy K.M., Chabo C. (2007). Metabolic endotoxemia initiates obesity and insulin resistance. Diabetes.

[23] Rivière A., Selak M., Lantin D., Leroy F., De Vuyst L. (2016). Bifidobacteria and Butyrate-Producing Colon Bacteria: Importance and Strategies for Their Stimulation in the Human Gut. Front. Microbiol..

[24] Müller T.D., Finan B., Bloom S.R., D’Alessio D., Drucker D.J., Flatt P.R., Fritsche A., Gribble F., Grill H.J., Habener J.F. (2019). Glucagon-like peptide 1 (GLP-1). Mol. Metab..

[25] van Bloemendaal L., Veltman D.J., Ten Kulve J.S., Groot P.F., Ruhé H.G., Barkhof F., Sloan J.H., Diamant M., Ijzerman R.G. (2015). Brain reward-system activation in response to anticipation and consumption of palatable food is altered by glucagon-like peptide-1 receptor activation in humans. Diabetes Obes. Metab..

[26] Salvatore T., Nevola R., Pafundi P.C., Monaco L., Ricozzi C., Imbriani S., Rinaldi L., Sasso F.C. (2019). Incretin Hormones: The Link between Glycemic Index and Cardiometabolic Diseases. Nutrients.

[27] Hundt M., Basit H., John S. (2024). Physiology, Bile Secretion. StatPearls.

[28] Lucas L.N., Barrett K., Kerby R.L., Zhang Q., Cattaneo L.E., Stevenson D., Rey F.E., Amador-Noguez D. (2021). Dominant Bacterial Phyla from the Human Gut Show Widespread Ability To Transform and Conjugate Bile Acids. mSystems.

[29] Gropper S.S., Smith J.L. (2013). Advanced Nutrition and Human Metabolism.

[30] Ridlon J.M., Kang D.J., Hylemon P.B. (2010). Isolation and characterization of a bile acid inducible 7alpha-dehydroxylating operon in *Clostridium hylemonae* TN271. Anaerobe.

[31] Wan Y.Y., Sheng L. (2018). Regulation of bile acid receptor activity. Liver Res..

[32] Chand D., Ramasamy S., Suresh C.G. (2016). A highly active bile salt hydrolase from Enterococcus faecalis shows positive cooperative kinetics. Process Biochem..

[33] Doden H., Sallam L.A., Devendran S., Ly L., Doden G., Daniel S.L., Alves J.M.P., Ridlon J.M. (2018). Metabolism of Oxo-Bile Acids and Characterization of Recombinant 12α-Hydroxysteroid Dehydrogenases from Bile Acid 7α-Dehydroxylating Human Gut Bacteria. Appl. Environ. Microbiol..

[34] Gérard P. (2013). Metabolism of cholesterol and bile acids by the gut microbiota. Pathogens.

[35] Sato Y., Atarashi K., Plichta D.R., Arai Y., Sasajima S., Kearney S.M., Suda W., Takeshita K., Sasaki T., Okamoto S. (2021). Novel bile acid biosynthetic pathways are enriched in the microbiome of centenarians. Nature.

[36] Kiriyama Y., Nochi H. (2023). The Role of Gut Microbiota-Derived Lithocholic Acid, Deoxycholic Acid and Their Derivatives on the Function and Differentiation of Immune Cells. Microorganisms.

[37] Hao Y., Han L., Wu A., Bochkis I.M. (2024). Pioneer Factor Foxa2 Mediates Chromatin Conformation Changes for Activation of Bile Acid Targets of FXR. Cell Mol. Gastroenterol. Hepatol..

[38] Liu S., Chen X., He J., Luo Y., Zheng P., Yu B., Chen D., Huang Z. (2024). Oleanolic acid promotes skeletal muscle fiber type transformation by activating TGR5-mediated CaN signaling pathway. J. Nutr. Biochem..

[39] Schertzer J.D., Lam T.K.T. (2021). Peripheral and central regulation of insulin by the intestine and microbiome. Am. J. Physiol. Endocrinol. Metab..

[40] Inagaki T., Moschetta A., Lee Y.-K., Peng L., Zhao G., Downes M., Yu R.T., Shelton J.M., Richardson J.A., Repa J.J. (2006). Regulation of antibacterial defense in the small intestine by the nuclear bile acid receptor. Proc. Natl. Acad. Sci. USA.

[41] Wahlström A., Sayin S.I., Marschall H.U., Bäckhed F. (2016). Intestinal Crosstalk between Bile Acids and Microbiota and Its Impact on Host Metabolism. Cell Metab..

[42] Kasprzak-Drozd K., Oniszczuk T., Stasiak M., Oniszczuk A. (2021). Beneficial Effects of Phenolic Compounds on Gut Microbiota and Metabolic Syndrome. Int. J. Mol. Sci..

[43] Pereira L., Valado A. (2023). Algae-Derived Natural Products in Diabetes and Its Complications—Current Advances and Future Prospects. Life.

[44] Wu Q., Sun L., Hu X., Wang X., Xu F., Chen B., Liang X., Xia J., Wang P., Aibara D. (2021). Suppressing the intestinal farnesoid X receptor/sphingomyelin phosphodiesterase 3 axis decreases atherosclerosis. J. Clin. Investig..

[45] Nallathambi R., Poulev A., Zuk J.B., Raskin I. (2020). Proanthocyanidin-Rich Grape Seed Extract Reduces Inflammation and Oxidative Stress and Restores Tight Junction Barrier Function in Caco-2 Colon Cells. Nutrients.

[46] Moszak M., Szulińska M., Bogdański P. (2020). You Are What You Eat-The Relationship between Diet, Microbiota, and Metabolic Disorders-A Review. Nutrients.

[47] Harper A., Vijayakumar V., Ouwehand A.C., Ter Haar J., Obis D., Espadaler J., Binda S., Desiraju S., Day R. (2020). Viral Infections, the Microbiome, and Probiotics. Front. Cell Infect. Microbiol..

[48] Karakan T., Ozkul C., Küpeli Akkol E., Bilici S., Sobarzo-Sánchez E., Capasso R. (2021). Gut-Brain-Microbiota Axis: Antibiotics and Functional Gastrointestinal Disorders. Nutrients.

[49] Martel J., Chang S.H., Ko Y.F., Hwang T.L., Young J.D., Ojcius D.M. (2022). Gut barrier disruption and chronic disease. Trends Endocrinol. Metab..

[50] Miao S., Qiu H. (2024). The microbiome in the pathogenesis of lung cancer: The role of microbiome in lung cancer pathogenesis. Apmis.

[51] Marzhoseyni Z., Shaghaghi Z., Alvandi M., Shirvani M. (2024). Investigating the Influence of Gut Microbiota-related Metabolites in Gastrointestinal Cancer. Curr. Cancer Drug Targets.

[52] Rajapakse J., Khatiwada S., Akon A.C., Yu K.L., Shen S., Zekry A. (2023). Unveiling the complex relationship between gut microbiota and liver cancer: Opportunities for novel therapeutic interventions. Gut Microbes.

[53] Pourali G., Kazemi D., Chadeganipour A.S., Arastonejad M., Kashani S.N., Pourali R., Maftooh M., Akbarzade H., Fiuji H., Hassanian S.M. (2024). Microbiome as a biomarker and therapeutic target in pancreatic cancer. BMC Microbiol..

[54] Chen J., Vitetta L. (2019). Bile acids and butyrate in the effects of probiotics/synbiotics on nonalcoholic fatty liver disease. Eur. J. Gastroenterol. Hepatol..

[55] Chen J., Vitetta L. (2020). Mitochondria could be a potential key mediator linking the intestinal microbiota to depression. J. Cell Biochem..

[56] Kocot A.M., Jarocka-Cyrta E., Drabińska N. (2022). Overview of the Importance of Biotics in Gut Barrier Integrity. Int. J. Mol. Sci..

[57] Soares J.B., Pimentel-Nunes P., Roncon-Albuquerque R., Leite-Moreira A. (2010). The role of lipopolysaccharide/toll-like receptor 4 signaling in chronic liver diseases. Hepatol. Int..

[58] Kessoku T., Kobayashi T., Tanaka K., Yamamoto A., Takahashi K., Iwaki M., Ozaki A., Kasai Y., Nogami A., Honda Y. (2021). The Role of Leaky Gut in Nonalcoholic Fatty Liver Disease: A Novel Therapeutic Target. Int. J. Mol. Sci..

[59] Baumann A., Nier A., Hernández-Arriaga A., Brandt A., Lorenzo Pisarello M.J., Jin C.J., Pilar E., Camarinha-Silva A., Schattenberg J.M., Bergheim I. (2021). Toll-like receptor 1 as a possible target in non-alcoholic fatty liver disease. Sci. Rep..

[60] Loomba R., Friedman S.L., Shulman G.I. (2021). Mechanisms and disease consequences of nonalcoholic fatty liver disease. Cell.

[61] Pierantonelli I., Svegliati-Baroni G. (2019). Nonalcoholic Fatty Liver Disease: Basic Pathogenetic Mechanisms in the Progression From NAFLD to NASH. Transplantation.

[62] Gabbia D., Cannella L., De Martin S. (2021). The Role of Oxidative Stress in NAFLD-NASH-HCC Transition-Focus on NADPH Oxidases. Biomedicines.

[63] Boursier J., Mueller O., Barret M., Machado M., Fizanne L., Araujo-Perez F., Guy C.D., Seed P.C., Rawls J.F., David L.A. (2016). The severity of nonalcoholic fatty liver disease is associated with gut dysbiosis and shift in the metabolic function of the gut microbiota. Hepatology.

[64] Magne F., Gotteland M., Gauthier L., Zazueta A., Pesoa S., Navarrete P., Balamurugan R. (2020). The Firmicutes/Bacteroidetes Ratio: A Relevant Marker of Gut Dysbiosis in Obese Patients?. Nutrients.

[65] de Wit N., Derrien M., Bosch-Vermeulen H., Oosterink E., Keshtkar S., Duval C., de Vogel-van den Bosch J., Kleerebezem M., Müller M., van der Meer R. (2012). Saturated fat stimulates obesity and hepatic steatosis and affects gut microbiota composition by an enhanced overflow of dietary fat to the distal intestine. Am. J. Physiol. Gastrointest. Liver Physiol..

[66] Hildebrandt M.A., Hoffmann C., Sherrill-Mix S.A., Keilbaugh S.A., Hamady M., Chen Y.Y., Knight R., Ahima R.S., Bushman F., Wu G.D. (2009). High-fat diet determines the composition of the murine gut microbiome independently of obesity. Gastroenterology.

[67] Aron-Wisnewsky J., Vigliotti C., Witjes J., Le P., Holleboom A.G., Verheij J., Nieuwdorp M., Clément K. (2020). Gut microbiota and human NAFLD: Disentangling microbial signatures from metabolic disorders. Nat. Rev. Gastroenterol. Hepatol..

[68] Peng Y., Lin H., Tian S., Liu S., Li J., Lv X., Chen S., Zhao L., Pu F., Chen X. (2021). Glucagon-like peptide-1 receptor activation maintains extracellular matrix integrity by inhibiting the activity of mitogen-activated protein kinases and activator protein-1. Free Radic. Biol. Med..

[69] Louis P., Flint H.J. (2017). Formation of propionate and butyrate by the human colonic microbiota. Environ. Microbiol..

[70] Cummings J.H., Pomare E.W., Branch W.J., Naylor C.P., Macfarlane G.T. (1987). Short chain fatty acids in human large intestine, portal, hepatic and venous blood. Gut.

[71] Vogt J.A., Pencharz P.B., Wolever T.M. (2004). L-Rhamnose increases serum propionate in humans. Am. J. Clin. Nutr..

[72] Nilsson A.C., Östman E.M., Knudsen K.E., Holst J.J., Björck I.M. (2010). A cereal-based evening meal rich in indigestible carbohydrates increases plasma butyrate the next morning. J. Nutr..

[73] Le Poul E., Loison C., Struyf S., Springael J.Y., Lannoy V., Decobecq M.E., Brezillon S., Dupriez V., Vassart G., Van Damme J. (2003). Functional characterization of human receptors for short chain fatty acids and their role in polymorphonuclear cell activation. J. Biol. Chem..

[74] Kimura T., Pydi S.P., Pham J., Tanaka N. (2020). Metabolic Functions of G Protein-Coupled Receptors in Hepatocytes-Potential Applications for Diabetes and NAFLD. Biomolecules.

[75] Zhou D., Chen Y.W., Zhao Z.H., Yang R.X., Xin F.Z., Liu X.L., Pan Q., Zhou H., Fan J.G. (2018). Sodium butyrate reduces high-fat diet-induced non-alcoholic steatohepatitis through upregulation of hepatic GLP-1R expression. Exp. Mol. Med..

[76] Dubois V., Eeckhoute J., Lefebvre P., Staels B. (2017). Distinct but complementary contributions of PPAR isotypes to energy homeostasis. J. Clin. Investig..

[77] Lange N.F., Graf V., Caussy C., Dufour J.F. (2022). PPAR-Targeted Therapies in the Treatment of Non-Alcoholic Fatty Liver Disease in Diabetic Patients. Int. J. Mol. Sci..

[78] Xu X., Poulsen K.L., Wu L., Liu S., Miyata T., Song Q., Wei Q., Zhao C., Lin C., Yang J. (2022). Targeted therapeutics and novel signaling pathways in non-alcohol-associated fatty liver/steatohepatitis (NAFL/NASH). Signal Transduct. Target. Ther..

[79] Liu L., Fu Q., Li T., Shao K., Zhu X., Cong Y., Zhao X. (2022). Gut microbiota and butyrate contribute to nonalcoholic fatty liver disease in premenopause due to estrogen deficiency. PLoS ONE.

[80] Byndloss M.X., Olsan E.E., Rivera-Chávez F., Tiffany C.R., Cevallos S.A., Lokken K.L., Torres T.P., Byndloss A.J., Faber F., Gao Y. (2017). Microbiota-activated PPAR-γ signaling inhibits dysbiotic Enterobacteriaceae expansion. Science.

[81] Wu L., Li J., Feng J., Ji J., Yu Q., Li Y., Zheng Y., Dai W., Wu J., Guo C. (2021). Crosstalk between PPARs and gut microbiota in NAFLD. Biomed. Pharmacother..

[82] Kondo T., Kishi M., Fushimi T., Kaga T. (2009). Acetic acid upregulates the expression of genes for fatty acid oxidation enzymes in liver to suppress body fat accumulation. J. Agric. Food Chem..

[83] Duarte S.M.B., Stefano J.T., Oliveira C.P. (2019). Microbiota and nonalcoholic fatty liver disease/nonalcoholic steatohepatitis (NAFLD/NASH). Ann. Hepatol..

[84] Hong J., Jia Y., Pan S., Jia L., Li H., Han Z., Cai D., Zhao R. (2016). Butyrate alleviates high fat diet-induced obesity through activation of adiponectin-mediated pathway and stimulation of mitochondrial function in the skeletal muscle of mice. Oncotarget.

[85] Wu J.L., Zou J.Y., Hu E.D., Chen D.Z., Chen L., Lu F.B., Xu L.M., Zheng M.H., Li H., Huang Y. (2017). Sodium butyrate ameliorates S100/FCA-induced autoimmune hepatitis through regulation of intestinal tight junction and toll-like receptor 4 signaling pathway. Immunol. Lett..

[86] Hu E.D., Chen D.Z., Wu J.L., Lu F.B., Chen L., Zheng M.H., Li H., Huang Y., Li J., Jin X.Y. (2018). High fiber dietary and sodium butyrate attenuate experimental autoimmune hepatitis through regulation of immune regulatory cells and intestinal barrier. Cell Immunol..

[87] Deng M., Qu F., Chen L., Liu C., Zhang M., Ren F., Guo H., Zhang H., Ge S., Wu C. (2020). SCFAs alleviated steatosis and inflammation in mice with NASH induced by MCD. J. Endocrinol..

[88] Korsten S., Vromans H., Garssen J., Willemsen L.E.M. (2023). Butyrate Protects Barrier Integrity and Suppresses Immune Activation in a Caco-2/PBMC Co-Culture Model While HDAC Inhibition Mimics Butyrate in Restoring Cytokine-Induced Barrier Disruption. Nutrients.

[89] Armstrong M.J., Hull D., Guo K., Barton D., Hazlehurst J.M., Gathercole L.L., Nasiri M., Yu J., Gough S.C., Newsome P.N. (2016). Glucagon-like peptide 1 decreases lipotoxicity in non-alcoholic steatohepatitis. J. Hepatol..

[90] Bullock B.P., Heller R.S., Habener J.F. (1996). Tissue distribution of messenger ribonucleic acid encoding the rat glucagon-like peptide-1 receptor. Endocrinology.

[91] Campos R.V., Lee Y.C., Drucker D.J. (1994). Divergent tissue-specific and developmental expression of receptors for glucagon and glucagon-like peptide-1 in the mouse. Endocrinology.

[92] Cusi K. (2016). Treatment of patients with type 2 diabetes and non-alcoholic fatty liver disease: Current approaches and future directions. Diabetologia.

[93] Marchesini G., Brizi M., Bianchi G., Tomassetti S., Bugianesi E., Lenzi M., McCullough A.J., Natale S., Forlani G., Melchionda N. (2001). Nonalcoholic fatty liver disease: A feature of the metabolic syndrome. Diabetes.

[94] Ding X., Saxena N.K., Lin S., Gupta N.A., Anania F.A. (2006). Exendin-4, a glucagon-like protein-1 (GLP-1) receptor agonist, reverses hepatic steatosis in ob/ob mice. Hepatology.

[95] Zhang L., Yang M., Ren H., Hu H., Boden G., Li L., Yang G. (2013). GLP-1 analogue prevents NAFLD in ApoE KO mice with diet and Acrp30 knockdown by inhibiting c-JNK. Liver Int..

[96] Kaji I., Karaki S., Kuwahara A. (2014). Short-chain fatty acid receptor and its contribution to glucagon-like peptide-1 release. Digestion.

[97] Muscogiuri G., DeFronzo R.A., Gastaldelli A., Holst J.J. (2017). Glucagon-like Peptide-1 and the Central/Peripheral Nervous System: Crosstalk in Diabetes. Trends Endocrinol. Metab..

[98] Samson S.L., Sathyanarayana P., Jogi M., Gonzalez E.V., Gutierrez A., Krishnamurthy R., Muthupillai R., Chan L., Bajaj M. (2011). Exenatide decreases hepatic fibroblast growth factor 21 resistance in non-alcoholic fatty liver disease in a mouse model of obesity and in a randomised controlled trial. Diabetologia.

[99] Cusi K., Sattar N., García-Pérez L.E., Pavo I., Yu M., Robertson K.E., Karanikas C.A., Haupt A. (2018). Dulaglutide decreases plasma aminotransferases in people with Type 2 diabetes in a pattern consistent with liver fat reduction: A post hoc analysis of the AWARD programme. Diabet. Med..

[100] Armstrong M.J., Gaunt P., Aithal G.P., Barton D., Hull D., Parker R., Hazlehurst J.M., Guo K., Abouda G., Aldersley M.A. (2016). Liraglutide safety and efficacy in patients with non-alcoholic steatohepatitis (LEAN): A multicentre, double-blind, randomised, placebo-controlled phase 2 study. Lancet.

[101] Chen J., Thomsen M., Vitetta L. (2019). Interaction of gut microbiota with dysregulation of bile acids in the pathogenesis of nonalcoholic fatty liver disease and potential therapeutic implications of probiotics. J. Cell Biochem..

[102] Wang H., Chen J., Hollister K., Sowers L.C., Forman B.M. (1999). Endogenous bile acids are ligands for the nuclear receptor FXR/BAR. Mol. Cell.

[103] Pols T.W., Noriega L.G., Nomura M., Auwerx J., Schoonjans K. (2011). The bile acid membrane receptor TGR5: A valuable metabolic target. Dig. Dis..

[104] Sinal C.J., Tohkin M., Miyata M., Ward J.M., Lambert G., Gonzalez F.J. (2000). Targeted disruption of the nuclear receptor FXR/BAR impairs bile acid and lipid homeostasis. Cell.

[105] Xi Y., Li H. (2020). Role of farnesoid X receptor in hepatic steatosis in nonalcoholic fatty liver disease. Biomed. Pharmacother..

[106] Kong B., Luyendyk J.P., Tawfik O., Guo G.L. (2009). Farnesoid X receptor deficiency induces nonalcoholic steatohepatitis in low-density lipoprotein receptor-knockout mice fed a high-fat diet. J. Pharmacol. Exp. Ther..

[107] Verbeke L., Mannaerts I., Schierwagen R., Govaere O., Klein S., Vander Elst I., Windmolders P., Farre R., Wenes M., Mazzone M. (2016). FXR agonist obeticholic acid reduces hepatic inflammation and fibrosis in a rat model of toxic cirrhosis. Sci. Rep..

[108] Watanabe M., Houten S.M., Wang L., Moschetta A., Mangelsdorf D.J., Heyman R.A., Moore D.D., Auwerx J. (2004). Bile acids lower triglyceride levels via a pathway involving FXR, SHP, and SREBP-1c. J. Clin. Investig..

[109] Thorsson V., Gibbs D.L., Brown S.D., Wolf D., Bortone D.S., Ou Yang T.-H., Porta-Pardo E., Gao G.F., Plaisier C.L., Eddy J.A. (2018). The Immune Landscape of Cancer. Immunity.

[110] Schumacher J.D., Kong B., Wu J., Rizzolo D., Armstrong L.E., Chow M.D., Goedken M., Lee Y.H., Guo G.L. (2020). Direct and Indirect Effects of Fibroblast Growth Factor (FGF) 15 and FGF19 on Liver Fibrosis Development. Hepatology.

[111] Adorini L., Trauner M. (2023). FXR agonists in NASH treatment. J. Hepatol..

[112] Thomas C., Gioiello A., Noriega L., Strehle A., Oury J., Rizzo G., Macchiarulo A., Yamamoto H., Mataki C., Pruzanski M. (2009). TGR5-mediated bile acid sensing controls glucose homeostasis. Cell Metab..

[113] Vassileva G., Hu W., Hoos L., Tetzloff G., Yang S., Liu L., Kang L., Davis H.R., Hedrick J.A., Lan H. (2010). Gender-dependent effect of Gpbar1 genetic deletion on the metabolic profiles of diet-induced obese mice. J. Endocrinol..

[114] Baffy G. (2009). Kupffer cells in non-alcoholic fatty liver disease: The emerging view. J. Hepatol..

[115] Shi Y., Su W., Zhang L., Shi C., Zhou J., Wang P., Wang H., Shi X., Wei S., Wang Q. (2020). TGR5 Regulates Macrophage Inflammation in Nonalcoholic Steatohepatitis by Modulating NLRP3 Inflammasome Activation. Front. Immunol..

[116] Jia W., Xie G. (2018). Bile acid-microbiota crosstalk in gastrointestinal inflammation and carcinogenesis. Nat. Rev. Gastroenterol. Hepatol..

[117] Hardwick R.N., Ferreira D.W., More V.R., Lake A.D., Lu Z., Manautou J.E., Slitt A.L., Cherrington N.J. (2013). Altered UDP-glucuronosyltransferase and sulfotransferase expression and function during progressive stages of human nonalcoholic fatty liver disease. Drug Metab. Dispos..

[118] Qin J., Li Y., Cai Z., Li S., Zhu J., Zhang F., Liang S., Zhang W., Guan Y., Shen D. (2012). A metagenome-wide association study of gut microbiota in type 2 diabetes. Nature.

[119] Brown A.J., Goldsworthy S.M., Barnes A.A., Eilert M.M., Tcheang L., Daniels D., Muir A.I., Wigglesworth M.J., Kinghorn I., Fraser N.J. (2003). The Orphan G protein-coupled receptors GPR41 and GPR43 are activated by propionate and other short chain carboxylic acids. J. Biol. Chem..

[120] Tang C., Ahmed K., Gille A., Lu S., Gröne H.J., Tunaru S., Offermanns S. (2015). Loss of FFA2 and FFA3 increases insulin secretion and improves glucose tolerance in type 2 diabetes. Nat. Med..

[121] McNelis J.C., Lee Y.S., Mayoral R., van der Kant R., Johnson A.M., Wollam J., Olefsky J.M. (2015). GPR43 Potentiates β-Cell Function in Obesity. Diabetes.

[122] van de Wouw M., Boehme M., Lyte J.M., Wiley N., Strain C., O’Sullivan O., Clarke G., Stanton C., Dinan T.G., Cryan J.F. (2018). Short-chain fatty acids: Microbial metabolites that alleviate stress-induced brain-gut axis alterations. J. Physiol..

[123] Kowluru A. (2003). Regulatory roles for small G proteins in the pancreatic beta-cell: Lessons from models of impaired insulin secretion. Am. J. Physiol. Endocrinol. Metab..

[124] Veprik A., Laufer D., Weiss S., Rubins N., Walker M.D. (2016). GPR41 modulates insulin secretion and gene expression in pancreatic β-cells and modifies metabolic homeostasis in fed and fasting states. Faseb J..

[125] Yamaguchi Y., Adachi K., Sugiyama T., Shimozato A., Ebi M., Ogasawara N., Funaki Y., Goto C., Sasaki M., Kasugai K. (2016). Association of Intestinal Microbiota with Metabolic Markers and Dietary Habits in Patients with Type 2 Diabetes. Digestion.

[126] Li L., Pan M., Pan S., Li W., Zhong Y., Hu J., Nie S. (2020). Effects of insoluble and soluble fibers isolated from barley on blood glucose, serum lipids, liver function and caecal short-chain fatty acids in type 2 diabetic and normal rats. Food Chem. Toxicol..

[127] Mandaliya D.K., Seshadri S. (2019). Short Chain Fatty Acids, pancreatic dysfunction and type 2 diabetes. Pancreatology.

[128] Zheng J., An Y., Du Y., Song Y., Zhao Q., Lu Y. (2024). Effects of short-chain fatty acids on blood glucose and lipid levels in mouse models of diabetes mellitus: A systematic review and network meta-analysis. Pharmacol. Res..

[129] Matheus V.A., Monteiro L., Oliveira R.B., Maschio D.A., Collares-Buzato C.B. (2017). Butyrate reduces high-fat diet-induced metabolic alterations, hepatic steatosis and pancreatic beta cell and intestinal barrier dysfunctions in prediabetic mice. Exp. Biol. Med..

[130] Li J., Li Y., Zhang S., Wang C., Mao Z., Huo W., Yang T., Xing W., Li L. (2024). Association of the short-chain fatty acid levels and dietary quality with type 2 diabetes: A case-control study based on Henan Rural Cohort. Br. J. Nutr..

[131] Drucker D.J. (2006). The biology of incretin hormones. Cell Metab..

[132] Saad M.J., Santos A., Prada P.O. (2016). Linking Gut Microbiota and Inflammation to Obesity and Insulin Resistance. Physiology.

[133] Lee J.Y., Bae E., Kim H.Y., Lee K.M., Yoon S.S., Lee D.C. (2021). High-Fat-Diet-Induced Oxidative Stress Linked to the Increased Colonization of *Lactobacillus sakei* in an Obese Population. Microbiol. Spectr..

[134] Onoviran O.F., Li D., Toombs Smith S., Raji M.A. (2019). Effects of glucagon-like peptide 1 receptor agonists on comorbidities in older patients with diabetes mellitus. Ther. Adv. Chronic Dis..

[135] Nauck M.A., D’Alessio D.A. (2022). Tirzepatide, a dual GIP/GLP-1 receptor co-agonist for the treatment of type 2 diabetes with unmatched effectiveness regrading glycaemic control and body weight reduction. Cardiovasc. Diabetol..

[136] Ordóñez-Vázquez A.L., Beltrán-Gall S.M., Pal S.C., Méndez-Sánchez N. (2022). Editorial: Treatment with Dual Incretin Receptor Agonists to Maintain Normal Glucose Levels May Also Maintain Normal Weight and Control Metabolic Dysfunction-Associated Fatty Liver Disease (MAFLD). Med. Sci. Monit..

[137] Holst J.J., McGill M.A. (2012). Potential New Approaches to Modifying Intestinal GLP-1 Secretion in Patients with Type 2 Diabetes Mellitus: Focus on Bile Acid Sequestrants. Clin. Drug Investig..

[138] Maida A., Lamont B.J., Cao X., Drucker D.J. (2011). Metformin regulates the incretin receptor axis via a pathway dependent on peroxisome proliferator-activated receptor-α in mice. Diabetologia.

[139] Ma Q., Li Y., Li P., Wang M., Wang J., Tang Z., Wang T., Luo L., Wang C., Zhao B. (2019). Research progress in the relationship between type 2 diabetes mellitus and intestinal flora. Biomed. Pharmacother..

[140] Zhang L., Wu W., Lee Y.K., Xie J., Zhang H. (2018). Spatial Heterogeneity and Co-occurrence of Mucosal and Luminal Microbiome across Swine Intestinal Tract. Front. Microbiol..

[141] Chávez-Talavera O., Wargny M., Pichelin M., Descat A., Vallez E., Kouach M., Bigot-Corbel E., Joliveau M., Goossens J.F., Le May C. (2020). Bile acids associate with glucose metabolism, but do not predict conversion from impaired fasting glucose to diabetes. Metabolism.

[142] Castañeda T.R., Méndez M., Davison I., Elvert R., Schwahn U., Boldina G., Rocher C., Scherer P., Singh K., Bangari D.S. (2021). The Novel Phosphate and Bile Acid Sequestrant Polymer SAR442357 Delays Disease Progression in a Rat Model of Diabetic Nephropathy. J. Pharmacol. Exp. Ther..

[143] Cipriani S., Mencarelli A., Chini M.G., Distrutti E., Renga B., Bifulco G., Baldelli F., Donini A., Fiorucci S. (2011). The bile acid receptor GPBAR-1 (TGR5) modulates integrity of intestinal barrier and immune response to experimental colitis. PLoS ONE.

[144] Cariou B., van Harmelen K., Duran-Sandoval D., van Dijk T.H., Grefhorst A., Abdelkarim M., Caron S., Torpier G., Fruchart J.C., Gonzalez F.J. (2006). The farnesoid X receptor modulates adiposity and peripheral insulin sensitivity in mice. J. Biol. Chem..

[145] Cipriani S., Mencarelli A., Palladino G., Fiorucci S. (2010). FXR activation reverses insulin resistance and lipid abnormalities and protects against liver steatosis in Zucker (fa/fa) obese rats. J. Lipid Res..

[146] Chen B., Bai Y., Tong F., Yan J., Zhang R., Zhong Y., Tan H., Ma X. (2023). Glycoursodeoxycholic acid regulates bile acids level and alters gut microbiota and glycolipid metabolism to attenuate diabetes. Gut Microbes..

[147] Shaham O., Wei R., Wang T.J., Ricciardi C., Lewis G.D., Vasan R.S., Carr S.A., Thadhani R., Gerszten R.E., Mootha V.K. (2008). Metabolic profiling of the human response to a glucose challenge reveals distinct axes of insulin sensitivity. Mol. Syst. Biol..

[148] Patti M.E., Houten S.M., Bianco A.C., Bernier R., Larsen P.R., Holst J.J., Badman M.K., Maratos-Flier E., Mun E.C., Pihlajamaki J. (2009). Serum bile acids are higher in humans with prior gastric bypass: Potential contribution to improved glucose and lipid metabolism. Obesity.

[149] Thomas C., Auwerx J., Schoonjans K. (2008). Bile acids and the membrane bile acid receptor TGR5--connecting nutrition and metabolism. Thyroid.

[150] Katsuma S., Hirasawa A., Tsujimoto G. (2005). Bile acids promote glucagon-like peptide-1 secretion through TGR5 in a murine enteroendocrine cell line STC-1. Biochem. Biophys. Res. Commun..

[151] Fiorucci S., Antonelli E., Rizzo G., Renga B., Mencarelli A., Riccardi L., Orlandi S., Pellicciari R., Morelli A. (2004). The nuclear receptor SHP mediates inhibition of hepatic stellate cells by FXR and protects against liver fibrosis. Gastroenterology.

[152] Leiss O., von Bergmann K. (1982). Different effects of chenodeoxycholic acid and ursodeoxycholic acid on serum lipoprotein concentrations in patients with radiolucent gallstones. Scand. J. Gastroenterol..

[153] Yang Z.D., Guo Y.S., Huang J.S., Gao Y.F., Peng F., Xu R.Y., Su H.H., Zhang P.J. (2021). Isomaltulose Exhibits Prebiotic Activity, and Modulates Gut Microbiota, the Production of Short Chain Fatty Acids, and Secondary Bile Acids in Rats. Molecules.

[154] Shimazu T., Hirschey M.D., Newman J., He W., Shirakawa K., Le Moan N., Grueter C.A., Lim H., Saunders L.R., Stevens R.D. (2013). Suppression of oxidative stress by β-hydroxybutyrate, an endogenous histone deacetylase inhibitor. Science.

[155] Ahmadian M., Suh J.M., Hah N., Liddle C., Atkins A.R., Downes M., Evans R.M. (2013). PPARγ signaling and metabolism: The good, the bad and the future. Nat. Med..

[156] Boccatonda A., Andreetto L., D’Ardes D., Cocco G., Rossi I., Vicari S., Schiavone C., Cipollone F., Guagnano M.T. (2023). From NAFLD to MAFLD: Definition, Pathophysiological Basis and Cardiovascular Implications. Biomedicines.

[157] Bechmann L.P., Hannivoort R.A., Gerken G., Hotamisligil G.S., Trauner M., Canbay A. (2012). The interaction of hepatic lipid and glucose metabolism in liver diseases. J. Hepatol..

[158] Sheka A.C., Adeyi O., Thompson J., Hameed B., Crawford P.A., Ikramuddin S. (2020). Nonalcoholic Steatohepatitis: A Review. JAMA.

[159] Grzych G., Chávez-Talavera O., Descat A., Thuillier D., Verrijken A., Kouach M., Legry V., Verkindt H., Raverdy V., Legendre B. (2021). NASH-related increases in plasma bile acid levels depend on insulin resistance. JHEP Rep..

[160] De Vadder F., Kovatcheva-Datchary P., Goncalves D., Vinera J., Zitoun C., Duchampt A., Bäckhed F., Mithieux G. (2014). Microbiota-generated metabolites promote metabolic benefits via gut-brain neural circuits. Cell.

[161] Schneider K.M., Mohs A., Gui W., Galvez E.J.C., Candels L.S., Hoenicke L., Muthukumarasamy U., Holland C.H., Elfers C., Kilic K. (2022). Imbalanced gut microbiota fuels hepatocellular carcinoma development by shaping the hepatic inflammatory microenvironment. Nat. Commun..

[162] Weng M.T., Chiu Y.T., Wei P.Y., Chiang C.W., Fang H.L., Wei S.C. (2019). Microbiota and gastrointestinal cancer. J. Formos. Med. Assoc..

[163] Schwabe R.F., Greten T.F. (2020). Gut microbiome in HCC-Mechanisms, diagnosis and therapy. J. Hepatol..

[164] Sanduzzi Zamparelli M., Rocco A., Compare D., Nardone G. (2017). The gut microbiota: A new potential driving force in liver cirrhosis and hepatocellular carcinoma. United Eur. Gastroenterol. J..

[165] Yoshimoto S., Loo T.M., Atarashi K., Kanda H., Sato S., Oyadomari S., Iwakura Y., Oshima K., Morita H., Hattori M. (2013). Obesity-induced gut microbial metabolite promotes liver cancer through senescence secretome. Nature.

[166] Dapito D.H., Mencin A., Gwak G.Y., Pradere J.P., Jang M.K., Mederacke I., Caviglia J.M., Khiabanian H., Adeyemi A., Bataller R. (2012). Promotion of hepatocellular carcinoma by the intestinal microbiota and TLR4. Cancer Cell.

[167] Wang H.G., Huang X.D., Shen P., Li L.R., Xue H.T., Ji G.Z. (2013). Anticancer effects of sodium butyrate on hepatocellular carcinoma cells in vitro. Int. J. Mol. Med..

[168] Coradini D., Zorzet S., Rossin R., Scarlata I., Pellizzaro C., Turrin C., Bello M., Cantoni S., Speranza A., Sava G. (2004). Inhibition of hepatocellular carcinomas in vitro and hepatic metastases in vivo in mice by the histone deacetylase inhibitor HA-But. Clin. Cancer Res..

[169] Coradini D., Speranza A. (2005). Histone deacetylase inhibitors for treatment of hepatocellular carcinoma. Acta Pharmacol. Sin..

[170] Burgess D.J. (2012). Metabolism: Warburg behind the butyrate paradox?. Nat. Rev. Cancer.

[171] Jiang W., Guo Q., Wu J., Guo B., Wang Y., Zhao S., Lou H., Yu X., Mei X., Wu C. (2012). Dual effects of sodium butyrate on hepatocellular carcinoma cells. Mol. Biol. Rep..

[172] Wakabayashi K., Saito H., Kaneko F., Nakamoto N., Tada S., Hibi T. (2005). Gene expression associated with the decrease in malignant phenotype of human liver cancer cells following stimulation with a histone deacetylase inhibitor. Int. J. Oncol..

[173] Song Q., Zhang X., Liu W., Wei H., Liang W., Zhou Y., Ding Y., Ji F., Ho-Kwan Cheung A., Wong N. (2023). Bifidobacterium pseudolongum-generated acetate suppresses non-alcoholic fatty liver disease-associated hepatocellular carcinoma. J. Hepatol..

[174] Kobayashi M., Mikami D., Uwada J., Yazawa T., Kamiyama K., Kimura H., Taniguchi T., Iwano M. (2018). A short-chain fatty acid, propionate, enhances the cytotoxic effect of cisplatin by modulating GPR41 signaling pathways in HepG2 cells. Oncotarget.

[175] Thomas C., Pellicciari R., Pruzanski M., Auwerx J., Schoonjans K. (2008). Targeting bile-acid signalling for metabolic diseases. Nat. Rev. Drug Discov..

[176] Nguyen P.T., Kanno K., Pham Q.T., Kikuchi Y., Kakimoto M., Kobayashi T., Otani Y., Kishikawa N., Miyauchi M., Arihiro K. (2020). Senescent hepatic stellate cells caused by deoxycholic acid modulates malignant behavior of hepatocellular carcinoma. J. Cancer Res. Clin. Oncol..

[177] Oyama K., Shiota G., Ito H., Murawaki Y., Kawasaki H. (2002). Reduction of hepatocarcinogenesis by ursodeoxycholic acid in rats. Carcinogenesis.

[178] Yang F., Huang X., Yi T., Yen Y., Moore D.D., Huang W. (2007). Spontaneous development of liver tumors in the absence of the bile acid receptor farnesoid X receptor. Cancer Res..

[179] Degirolamo C., Modica S., Vacca M., Di Tullio G., Morgano A., D’Orazio A., Kannisto K., Parini P., Moschetta A. (2015). Prevention of spontaneous hepatocarcinogenesis in farnesoid X receptor-null mice by intestinal-specific farnesoid X receptor reactivation. Hepatology.

[180] Han L.Y., Fan Y.C., Mu N.N., Gao S., Li F., Ji X.F., Dou C.Y., Wang K. (2014). Aberrant DNA methylation of G-protein-coupled bile acid receptor Gpbar1 (TGR5) is a potential biomarker for hepatitis B Virus associated hepatocellular carcinoma. Int. J. Med. Sci..

[181] Zhou M., Mok M.T., Sun H., Chan A.W., Huang Y., Cheng A.S., Xu G. (2017). The anti-diabetic drug exenatide, a glucagon-like peptide-1 receptor agonist, counteracts hepatocarcinogenesis through cAMP-PKA-EGFR-STAT3 axis. Oncogene.

[182] Harding J.L., Shaw J.E., Peeters A., Cartensen B., Magliano D.J. (2015). Cancer risk among people with type 1 and type 2 diabetes: Disentangling true associations, detection bias, and reverse causation. Diabetes Care.

[183] Kleeff J., Korc M., Apte M., La Vecchia C., Johnson C.D., Biankin A.V., Neale R.E., Tempero M., Tuveson D.A., Hruban R.H. (2016). Pancreatic cancer. Nat. Rev. Dis. Primers.

[184] Pushalkar S., Hundeyin M., Daley D., Zambirinis C.P., Kurz E., Mishra A., Mohan N., Aykut B., Usyk M., Torres L.E. (2018). The Pancreatic Cancer Microbiome Promotes Oncogenesis by Induction of Innate and Adaptive Immune Suppression. Cancer Discov..

[185] Geller L.T., Barzily-Rokni M., Danino T., Jonas O.H., Shental N., Nejman D., Gavert N., Zwang Y., Cooper Z.A., Shee K. (2017). Potential role of intratumor bacteria in mediating tumor resistance to the chemotherapeutic drug gemcitabine. Science.

[186] Ren Z., Jiang J., Xie H., Li A., Lu H., Xu S., Zhou L., Zhang H., Cui G., Chen X. (2017). Gut microbial profile analysis by MiSeq sequencing of pancreatic carcinoma patients in China. Oncotarget.

[187] Mirzaei R., Afaghi A., Babakhani S., Sohrabi M.R., Hosseini-Fard S.R., Babolhavaeji K., Khani Ali Akbari S., Yousefimashouf R., Karampoor S. (2021). Role of microbiota-derived short-chain fatty acids in cancer development and prevention. Biomed. Pharmacother..

[188] Gidekel Friedlander S.Y., Chu G.C., Snyder E.L., Girnius N., Dibelius G., Crowley D., Vasile E., DePinho R.A., Jacks T. (2009). Context-dependent transformation of adult pancreatic cells by oncogenic K-Ras. Cancer Cell.

[189] Huang H., Daniluk J., Liu Y., Chu J., Li Z., Ji B., Logsdon C.D. (2014). Oncogenic K-Ras requires activation for enhanced activity. Oncogene.

[190] Kanika G., Khan S., Jena G. (2015). Sodium Butyrate Ameliorates L-Arginine-Induced Pancreatitis and Associated Fibrosis in Wistar Rat: Role of Inflammation and Nitrosative Stress. J. Biochem. Mol. Toxicol..

[191] Mullins T.D., Kern H.F., Metzgar R.S. (1991). Ultrastructural differentiation of sodium butyrate-treated human pancreatic adenocarcinoma cell lines. Pancreas.

[192] Panebianco C., Villani A., Pisati F., Orsenigo F., Ulaszewska M., Latiano T.P., Potenza A., Andolfo A., Terracciano F., Tripodo C. (2022). Butyrate, a postbiotic of intestinal bacteria, affects pancreatic cancer and gemcitabine response in in vitro and in vivo models. Biomed. Pharmacother..

[193] Bülow R., Fitzner B., Sparmann G., Emmrich J., Liebe S., Jaster R. (2007). Antifibrogenic effects of histone deacetylase inhibitors on pancreatic stellate cells. Biochem. Pharmacol..

[194] Lührs H., Gerke T., Boxberger F., Backhaus K., Melcher R., Scheppach W., Menzel T. (2001). Butyrate inhibits interleukin-1-mediated nuclear factor-kappa B activation in human epithelial cells. Dig. Dis. Sci..

[195] Kim M.H., Kang S.G., Park J.H., Yanagisawa M., Kim C.H. (2013). Short-chain fatty acids activate GPR41 and GPR43 on intestinal epithelial cells to promote inflammatory responses in mice. Gastroenterology.

[196] Wang L., Zhou W., Zhong Y., Huo Y., Fan P., Zhan S., Xiao J., Jin X., Gou S., Yin T. (2017). Overexpression of G protein-coupled receptor GPR87 promotes pancreatic cancer aggressiveness and activates NF-κB signaling pathway. Mol. Cancer.

[197] Tao J., Cheema H., Kesh K., Dudeja V., Dawra R., Roy S. (2022). Chronic pancreatitis in a caerulein-induced mouse model is associated with an altered gut microbiome. Pancreatology.

[198] Rees D.O., Crick P.J., Jenkins G.J., Wang Y., Griffiths W.J., Brown T.H., Al-Sarireh B. (2017). Comparison of the composition of bile acids in bile of patients with adenocarcinoma of the pancreas and benign disease. J. Steroid Biochem. Mol. Biol..

[199] Gál E., Veréb Z., Kemény L., Rakk D., Szekeres A., Becskeházi E., Tiszlavicz L., Takács T., Czakó L., Hegyi P. (2020). Bile accelerates carcinogenic processes in pancreatic ductal adenocarcinoma cells through the overexpression of MUC4. Sci. Rep..

[200] Chen X.L., Xie K.X., Yang Z.L., Yuan L.W. (2019). Expression of FXR and HRG and their clinicopathological significance in benign and malignant pancreatic lesions. Int. J. Clin. Exp. Pathol..

[201] Lee J.Y., Lee K.T., Lee J.K., Lee K.H., Jang K.T., Heo J.S., Choi S.H., Kim Y., Rhee J.C. (2011). Farnesoid X receptor, overexpressed in pancreatic cancer with lymph node metastasis promotes cell migration and invasion. Br. J. Cancer.

[202] Sayin S.I., Wahlström A., Felin J., Jäntti S., Marschall H.U., Bamberg K., Angelin B., Hyötyläinen T., Orešič M., Bäckhed F. (2013). Gut microbiota regulates bile acid metabolism by reducing the levels of tauro-beta-muricholic acid, a naturally occurring FXR antagonist. Cell Metab..

[203] Nagathihalli N.S., Beesetty Y., Lee W., Washington M.K., Chen X., Lockhart A.C., Merchant N.B. (2014). Novel mechanistic insights into ectodomain shedding of EGFR Ligands Amphiregulin and TGF-α: Impact on gastrointestinal cancers driven by secondary bile acids. Cancer Res..

[204] Valerio A., Basso D., Brigato L., Ceolotto G., Baldo G., Tiengo A., Plebani M. (1999). Glucose metabolic alterations in isolated and perfused rat hepatocytes induced by pancreatic cancer conditioned medium: A low molecular weight factor possibly involved. Biochem. Biophys. Res. Commun..

[205] Cases A.I., Ohtsuka T., Kimura H., Zheng B., Shindo K., Oda Y., Mizumoto K., Nakamura M., Tanaka M. (2015). Significance of expression of glucagon-like peptide 1 receptor in pancreatic cancer. Oncol. Rep..

[206] Shlomai G., Neel B., LeRoith D., Gallagher E.J. (2016). Type 2 Diabetes Mellitus and Cancer: The Role of Pharmacotherapy. J. Clin. Oncol..

[207] Yu H., Rohan T. (2000). Role of the insulin-like growth factor family in cancer development and progression. J. Natl. Cancer Inst..

[208] Basso D., Valerio A., Brigato L., Panozzo M.P., Miola M., Lucca T., Ujka F., Zaninotto M., Avogaro A., Plebani M. (1997). An unidentified pancreatic cancer cell product alters some intracellular pathways of glucose metabolism in isolated rat hepatocytes. Pancreas.

[209] Koehler J.A., Drucker D.J. (2006). Activation of glucagon-like peptide-1 receptor signaling does not modify the growth or apoptosis of human pancreatic cancer cells. Diabetes.

[210] Zhao H., Jiang X., Duan L., Yang L., Wang W., Ren Z. (2019). Liraglutide suppresses the metastasis of PANC-1 co-cultured with pancreatic stellate cells through modulating intracellular calcium content. Endocr. J..

[211] Zhao H.J., Jiang X., Hu L.J., Yang L., Deng L.D., Wang Y.P., Ren Z.P. (2020). Activation of GLP-1 receptor enhances the chemosensitivity of pancreatic cancer cells. J. Mol. Endocrinol..

[212] Yan M., Shen M., Xu L., Huang J., He G., An M., Li X., Gao Z., Meng X. (2020). Inactivation of Pancreatic Stellate Cells by Exendin-4 Inhibits the Migration and Invasion of Pancreatic Cancer Cells. Onco Targets Ther..

[213] Zhang F., Luo W., Shi Y., Fan Z., Ji G. (2012). Should we standardize the 1700-year-old fecal microbiota transplantation?. Am. J. Gastroenterol..

[214] Borody T.J., Khoruts A. (2011). Fecal microbiota transplantation and emerging applications. Nat. Rev. Gastroenterol. Hepatol..

[215] Khoruts A., Sadowsky M.J., Hamilton M.J. (2015). Development of fecal microbiota transplantation suitable for mainstream medicine. Clin. Gastroenterol. Hepatol..

[216] Boicean A., Birlutiu V., Ichim C., Brusnic O., Onișor D.M. (2023). Fecal Microbiota Transplantation in Liver Cirrhosis. Biomedicines.

[217] Agrawal M., Aroniadis O.C., Brandt L.J., Kelly C., Freeman S., Surawicz C., Broussard E., Stollman N., Giovanelli A., Smith B. (2016). The Long-term Efficacy and Safety of Fecal Microbiota Transplant for Recurrent, Severe, and Complicated Clostridium difficile Infection in 146 Elderly Individuals. J. Clin. Gastroenterol..

[218] U.S. Food and Drug Administration (2013). Guidance for Industry: Enforcement Policy Regarding Investigational New Drug Requirements for Use of Fecal Microbiota for Transplantation To Treat Clostridium difficile Infection Not Responsive to Standard Therapies. Availability.

[219] Lin J., Xiong J., Jin Y., Wang H., Wu L., Chen L., Zhang F., Ji G., Cui B. (2024). Fecal microbiota transplantation through transendoscopic enteral tubing for inflammatory bowel disease: High acceptance and high satisfaction. J. Gastroenterol. Hepatol..

[220] Parigi T.L., Vieujean S., Paridaens K., Dalgaard K., Peyrin-Biroulet L., Danese S. (2023). Efficacy, Safety, and Concerns on Microbiota Modulation, Antibiotics, Probiotics, and Fecal Microbial Transplant for Inflammatory Bowel Disease and Other Gastrointestinal Conditions: Results from an International Survey. Microorganisms.

[221] Mandrioli J., Amedei A., Cammarota G., Niccolai E., Zucchi E., D’Amico R., Ricci F., Quaranta G., Spanu T., Masucci L. (2019). FETR-ALS Study Protocol: A Randomized Clinical Trial of Fecal Microbiota Transplantation in Amyotrophic Lateral Sclerosis. Front. Neurol..

[222] Martinelli S., Nannini G., Cianchi F., Staderini F., Coratti F., Amedei A. (2023). Microbiota Transplant and Gynecological Disorders: The Bridge between Present and Future Treatments. Microorganisms.

[223] Hege Marie H., Maria Serafia F., Linn S., Peter Holger J., Bård K., Rasmus G., Kristin Helen A., Per-Christian V. (2023). Randomised, placebo-controlled, double-blinded trial of fecal microbiota transplantation in severe obesity: A study protocol. BMJ Open.

[224] van Prehn J., Reigadas E., Vogelzang E.H., Bouza E., Hristea A., Guery B., Krutova M., Norén T., Allerberger F., Coia J.E. (2021). European Society of Clinical Microbiology and Infectious Diseases: 2021 update on the treatment guidance document for Clostridioides difficile infection in adults. Clin. Microbiol. Infect..

[225] Weingarden A.R., Chen C., Bobr A., Yao D., Lu Y., Nelson V.M., Sadowsky M.J., Khoruts A. (2014). Microbiota transplantation restores normal fecal bile acid composition in recurrent Clostridium difficile infection. Am. J. Physiol. Gastrointest. Liver Physiol..

[226] Bakken J.S., Borody T., Brandt L.J., Brill J.V., Demarco D.C., Franzos M.A., Kelly C., Khoruts A., Louie T., Martinelli L.P. (2011). Treating Clostridium difficile infection with fecal microbiota transplantation. Clin. Gastroenterol. Hepatol..

[227] Lee C.H., Steiner T., Petrof E.O., Smieja M., Roscoe D., Nematallah A., Weese J.S., Collins S., Moayyedi P., Crowther M. (2016). Frozen vs Fresh Fecal Microbiota Transplantation and Clinical Resolution of Diarrhea in Patients With Recurrent Clostridium difficile Infection: A Randomized Clinical Trial. JAMA.

[228] Vendrik K.E.W., Terveer E.M., Kuijper E.J., Nooij S., Boeije-Koppenol E., Sanders I., van Lingen E., Verspaget H.W., Berssenbrugge E.K.L., Keller J.J. (2021). Periodic screening of donor faeces with a quarantine period to prevent transmission of multidrug-resistant organisms during faecal microbiota transplantation: A retrospective cohort study. Lancet Infect. Dis..

[229] Porcari S., Benech N., Valles-Colomer M., Segata N., Gasbarrini A., Cammarota G., Sokol H., Ianiro G. (2023). Key determinants of success in fecal microbiota transplantation: From microbiome to clinic. Cell Host Microb..

[230] Wang J.W., Kuo C.H., Kuo F.C., Wang Y.K., Hsu W.H., Yu F.J., Hu H.M., Hsu P.I., Wang J.Y., Wu D.C. (2019). Fecal microbiota transplantation: Review and update. J. Formos. Med. Assoc..

[231] Janket S.J., Ackerson L.K., Diamandis E.P. (2020). Drug-Resistant Bacteremia after Fecal Microbiota Transplant. N. Engl. J. Med..

[232] Qu Z., Tian P., Yang B., Zhao J., Wang G., Chen W. (2022). Fecal microbiota transplantation for diseases: Therapeutic potential, methodology, risk management in clinical practice. Life Sci..

[233] Albillos A., de Gottardi A., Rescigno M. (2020). The gut-liver axis in liver disease: Pathophysiological basis for therapy. J. Hepatol..

[234] Scheithauer T.P.M., Rampanelli E., Nieuwdorp M., Vallance B.A., Verchere C.B., van Raalte D.H., Herrema H. (2020). Gut Microbiota as a Trigger for Metabolic Inflammation in Obesity and Type 2 Diabetes. Front. Immunol..

[235] Thaiss C.A., Levy M., Grosheva I., Zheng D., Soffer E., Blacher E., Braverman S., Tengeler A.C., Barak O., Elazar M. (2018). Hyperglycemia drives intestinal barrier dysfunction and risk for enteric infection. Science.

[236] Boursier J., Diehl A.M. (2015). Implication of gut microbiota in nonalcoholic fatty liver disease. PLoS Pathog..

[237] Llopis M., Cassard A.M., Wrzosek L., Boschat L., Bruneau A., Ferrere G., Puchois V., Martin J.C., Lepage P., Le Roy T. (2016). Intestinal microbiota contributes to individual susceptibility to alcoholic liver disease. Gut.

[238] Zhu L., Baker S.S., Gill C., Liu W., Alkhouri R., Baker R.D., Gill S.R. (2013). Characterization of gut microbiomes in nonalcoholic steatohepatitis (NASH) patients: A connection between endogenous alcohol and NASH. Hepatology.

[239] Del Chierico F., Nobili V., Vernocchi P., Russo A., De Stefanis C., Gnani D., Furlanello C., Zandonà A., Paci P., Capuani G. (2017). Gut microbiota profiling of pediatric nonalcoholic fatty liver disease and obese patients unveiled by an integrated meta-omics-based approach. Hepatology.

[240] Zhou X., Zhang X., Niu D., Zhang S., Wang H., Nan F., Jiang S., Wang B. (2023). Gut microbiota induces hepatic steatosis by modulating the T cells balance in high fructose diet mice. Sci. Rep..

[241] Shou D., Luo Q., Tang W., Cao C., Huang H., Chen H., Zhou Y. (2023). Hepatobiliary and pancreatic: Multi-donor fecal microbiota transplantation attenuated high-fat diet-induced hepatic steatosis in mice by remodeling the gut microbiota. J. Gastroenterol. Hepatol..

[242] Milton-Laskibar I., Cuevas-Sierra A., Portillo M.P., Martínez J.A. (2022). Effects of Resveratrol Administration in Liver Injury Prevention as Induced by an Obesogenic Diet: Role of Ruminococcaceae. Biomedicines.

[243] Chiu C.C., Ching Y.H., Li Y.P., Liu J.Y., Huang Y.T., Huang Y.W., Yang S.S., Huang W.C., Chuang H.L. (2017). Nonalcoholic Fatty Liver Disease Is Exacerbated in High-Fat Diet-Fed Gnotobiotic Mice by Colonization with the Gut Microbiota from Patients with Nonalcoholic Steatohepatitis. Nutrients.

[244] Zhou D., Pan Q., Shen F., Cao H.X., Ding W.J., Chen Y.W., Fan J.G. (2017). Total fecal microbiota transplantation alleviates high-fat diet-induced steatohepatitis in mice via beneficial regulation of gut microbiota. Sci. Rep..

[245] Lee D.H., Jee J.J., Lee Y.S., Kim D.Y., Bang J.Y., Lee H.W., Koh H., Bae S.H. (2023). Fecal microbiota transplantation improves hepatic fibro-inflammation via regulating oxidative stress in experimental NASH. Dig. Liver Dis..

[246] Loo T.M., Kamachi F., Watanabe Y., Yoshimoto S., Kanda H., Arai Y., Nakajima-Takagi Y., Iwama A., Koga T., Sugimoto Y. (2017). Gut Microbiota Promotes Obesity-Associated Liver Cancer through PGE(2)-Mediated Suppression of Antitumor Immunity. Cancer Discov..

[247] Ma C., Han M., Heinrich B., Fu Q., Zhang Q., Sandhu M., Agdashian D., Terabe M., Berzofsky J.A., Fako V. (2018). Gut microbiome-mediated bile acid metabolism regulates liver cancer via NKT cells. Science.

[248] Li Z., Zhang Y., Hong W., Wang B., Chen Y., Yang P., Zhou J., Fan J., Zeng Z., Du S. (2022). Gut microbiota modulate radiotherapy-associated antitumor immune responses against hepatocellular carcinoma Via STING signaling. Gut Microbes.

[249] Zhang X., Coker O.O., Chu E.S., Fu K., Lau H.C.H., Wang Y.X., Chan A.W.H., Wei H., Yang X., Sung J.J.Y. (2021). Dietary cholesterol drives fatty liver-associated liver cancer by modulating gut microbiota and metabolites. Gut.

[250] Das B.K. (2022). Altered gut microbiota in hepatocellular carcinoma: Insights into the pathogenic mechanism and preclinical to clinical findings. Apmis.

[251] Wang H., Lu Y., Yan Y., Tian S., Zheng D., Leng D., Wang C., Jiao J., Wang Z., Bai Y. (2019). Promising Treatment for Type 2 Diabetes: Fecal Microbiota Transplantation Reverses Insulin Resistance and Impaired Islets. Front. Cell Infect. Microbiol..

[252] Ding D., Yong H., You N., Lu W., Yang X., Ye X., Wang Y., Cai T., Zheng X., Chen H. (2022). Prospective Study Reveals Host Microbial Determinants of Clinical Response to Fecal Microbiota Transplant Therapy in Type 2 Diabetes Patients. Front. Cell Infect. Microbiol..

[253] Chen L., Guo L., Feng S., Wang C., Cui Z., Wang S., Lu Q., Chang H., Hang B., Snijders A.M. (2023). Fecal microbiota transplantation ameliorates type 2 diabetes via metabolic remodeling of the gut microbiota in db/db mice. BMJ Open Diabetes Res. Care.

[254] Yang W., Xia Z., Zhu Y., Tang H., Xu H., Hu X., Lin C., Jiang T., He P., Shen J. (2023). Comprehensive Study of Untargeted Metabolomics and 16S rRNA Reveals the Mechanism of Fecal Microbiota Transplantation in Improving a Mouse Model of T2D. Diabetes Metab. Syndr. Obes..

[255] Riquelme E., Zhang Y., Zhang L., Montiel M., Zoltan M., Dong W., Quesada P., Sahin I., Chandra V., San Lucas A. (2019). Tumor Microbiome Diversity and Composition Influence Pancreatic Cancer Outcomes. Cell.

[256] Tintelnot J., Xu Y., Lesker T.R., Schönlein M., Konczalla L., Giannou A.D., Pelczar P., Kylies D., Puelles V.G., Bielecka A.A. (2023). Microbiota-derived 3-IAA influences chemotherapy efficacy in pancreatic cancer. Nature.

[257] Qiu X.X., Cheng S.L., Liu Y.H., Li Y., Zhang R., Li N.N., Li Z. (2024). Fecal microbiota transplantation for treatment of non-alcoholic fatty liver disease: Mechanism, clinical evidence, and prospect. World J. Gastroenterol..

[258] Xue L., Deng Z., Luo W., He X., Chen Y. (2022). Effect of Fecal Microbiota Transplantation on Non-Alcoholic Fatty Liver Disease: A Randomized Clinical Trial. Front. Cell Infect. Microbiol..

[259] Craven L., Rahman A., Nair Parvathy S., Beaton M., Silverman J., Qumosani K., Hramiak I., Hegele R., Joy T., Meddings J. (2020). Allogenic Fecal Microbiota Transplantation in Patients With Nonalcoholic Fatty Liver Disease Improves Abnormal Small Intestinal Permeability: A Randomized Control Trial. Am. J. Gastroenterol..

[260] Bajaj J.S., Khoruts A. (2020). Microbiota changes and intestinal microbiota transplantation in liver diseases and cirrhosis. J. Hepatol..

[261] Bajaj J.S., Salzman N.H., Acharya C., Sterling R.K., White M.B., Gavis E.A., Fagan A., Hayward M., Holtz M.L., Matherly S. (2019). Fecal Microbial Transplant Capsules Are Safe in Hepatic Encephalopathy: A Phase 1, Randomized, Placebo-Controlled Trial. Hepatology.

[262] Ponziani F.R., Bhoori S., Castelli C., Putignani L., Rivoltini L., Del Chierico F., Sanguinetti M., Morelli D., Paroni Sterbini F., Petito V. (2019). Hepatocellular Carcinoma Is Associated With Gut Microbiota Profile and Inflammation in Nonalcoholic Fatty Liver Disease. Hepatology.

[263] Huang H., Ren Z., Gao X., Hu X., Zhou Y., Jiang J., Lu H., Yin S., Ji J., Zhou L. (2020). Integrated analysis of microbiome and host transcriptome reveals correlations between gut microbiota and clinical outcomes in HBV-related hepatocellular carcinoma. Genome Med..

[264] Li L., Ye J. (2020). Characterization of gut microbiota in patients with primary hepatocellular carcinoma received immune checkpoint inhibitors: A Chinese population-based study. Medicine.

[265] Chung M.W., Kim M.J., Won E.J., Lee Y.J., Yun Y.W., Cho S.B., Joo Y.E., Hwang J.E., Bae W.K., Chung I.J. (2021). Gut microbiome composition can predict the response to nivolumab in advanced hepatocellular carcinoma patients. World J. Gastroenterol..

[266] Zhao Y., Gong C., Xu J., Chen D., Yang B., Chen Z., Wei L. (2023). Research Progress of Fecal Microbiota Transplantation in Liver Diseases. J. Clin. Med..

[267] Wu Z., Zhang B., Chen F., Xia R., Zhu D., Chen B., Lin A., Zheng C., Hou D., Li X. (2022). Fecal microbiota transplantation reverses insulin resistance in type 2 diabetes: A randomized, controlled, prospective study. Front. Cell Infect. Microbiol..

[268] Ng S.C., Xu Z., Mak J.W.Y., Yang K., Liu Q., Zuo T., Tang W., Lau L., Lui R.N., Wong S.H. (2022). Microbiota engraftment after faecal microbiota transplantation in obese subjects with type 2 diabetes: A 24-week, double-blind, randomised controlled trial. Gut.

[269] Su L., Hong Z., Zhou T., Jian Y., Xu M., Zhang X., Zhu X., Wang J. (2022). Health improvements of type 2 diabetic patients through diet and diet plus fecal microbiota transplantation. Sci. Rep..

[270] Boicean A., Ichim C., Todor S.B., Anderco P., Popa M.L. (2024). The Importance of Microbiota and Fecal Microbiota Transplantation in Pancreatic Disorders. Diagnostics.

[271] Beam A., Clinger E., Hao L. (2021). Effect of Diet and Dietary Components on the Composition of the Gut Microbiota. Nutrients.

[272] van den Berg F.F., van Dalen D., Hyoju S.K., van Santvoort H.C., Besselink M.G., Wiersinga W.J., Zaborina O., Boermeester M.A., Alverdy J. (2021). Western-type diet influences mortality from necrotising pancreatitis and demonstrates a central role for butyrate. Gut.

[273] Yang D., Shen J., Tang C., Lu Z., Lu F., Bie X., Meng F., Zhao H. (2024). Prevention of high-fat-diet-induced obesity in mice by soluble dietary fiber from fermented and unfermented millet bran. Food Res. Int..

[274] Jaeger J.W., Brandt A., Gui W., Yergaliyev T., Hernández-Arriaga A., Muthu M.M., Edlund K., Elashy A., Molinaro A., Möckel D. (2024). Microbiota modulation by dietary oat beta-glucan prevents steatotic liver disease progression. JHEP Rep..

[275] Ghini V., Tenori L., Pane M., Amoruso A., Marroncini G., Squarzanti D.F., Azzimonti B., Rolla R., Savoia P., Tarocchi M. (2020). Effects of Probiotics Administration on Human Metabolic Phenotype. Metabolites.

[276] Singh S., Sharma R.K., Malhotra S., Pothuraju R., Shandilya U.K. (2017). *Lactobacillus rhamnosus* NCDC17 ameliorates type-2 diabetes by improving gut function, oxidative stress and inflammation in high-fat-diet fed and streptozotocintreated rats. Benef. Microbes.

[277] Yoo J.Y., Kim S.S. (2016). Probiotics and Prebiotics: Present Status and Future Perspectives on Metabolic Disorders. Nutrients.

[278] Wang W., Xu A.L., Li Z.C., Li Y., Xu S.F., Sang H.C., Zhi F. (2020). Combination of Probiotics and Salvia miltiorrhiza Polysaccharide Alleviates Hepatic Steatosis via Gut Microbiota Modulation and Insulin Resistance Improvement in High Fat-Induced NAFLD Mice. Diabetes Metab. J..

[279] Li H., Wang X.K., Tang M., Lei L., Li J.R., Sun H., Jiang J., Dong B., Li H.Y., Jiang J.D. (2024). Bacteroides thetaiotaomicron ameliorates mouse hepatic steatosis through regulating gut microbial composition, gut-liver folate and unsaturated fatty acids metabolism. Gut Microbes.

[280] Lau H.C., Zhang X., Ji F., Lin Y., Liang W., Li Q., Chen D., Fong W., Kang X., Liu W. (2024). *Lactobacillus acidophilus* suppresses non-alcoholic fatty liver disease-associated hepatocellular carcinoma through producing valeric acid. EBioMedicine.

[281] Koopen A., Witjes J., Wortelboer K., Majait S., Prodan A., Levin E., Herrema H., Winkelmeijer M., Aalvink S., Bergman J. (2022). Duodenal Anaerobutyricum soehngenii infusion stimulates GLP-1 production, ameliorates glycaemic control and beneficially shapes the duodenal transcriptome in metabolic syndrome subjects: A randomised double-blind placebo-controlled cross-over study. Gut.

[282] Sonnenburg J.L., Bäckhed F. (2016). Diet–microbiota interactions as moderators of human metabolism. Nature.

[283] Wang Y., Wen L., Tang H., Qu J., Rao B. (2023). Probiotics and Prebiotics as Dietary Supplements for the Adjunctive Treatment of Type 2 Diabetes. Pol. J. Microbiol..

[284] Sun Q., Spiegelman D., van Dam R.M., Holmes M.D., Malik V.S., Willett W.C., Hu F.B. (2010). White rice, brown rice, and risk of type 2 diabetes in US men and women. Arch. Intern. Med..

[285] Sun L., Xie C., Wang G., Wu Y., Wu Q., Wang X., Liu J., Deng Y., Xia J., Chen B. (2018). Gut microbiota and intestinal FXR mediate the clinical benefits of metformin. Nat. Med..

[286] Wang Y., Wu Y., Sailike J., Sun X., Abuduwaili N., Tuoliuhan H., Yusufu M., Nabi X.H. (2020). Fourteen composite probiotics alleviate type 2 diabetes through modulating gut microbiota and modifying M1/M2 phenotype macrophage in db/db mice. Pharmacol. Res..

[287] Wang Y., Dilidaxi D., Wu Y., Sailike J., Sun X., Nabi X.H. (2020). Composite probiotics alleviate type 2 diabetes by regulating intestinal microbiota and inducing GLP-1 secretion in db/db mice. Biomed. Pharmacother..

[288] Li H., Xie J., Guo X., Yang G., Cai B., Liu J., Yue M., Tang Y., Wang G., Chen S. (2022). Bifidobacterium spp. and their metabolite lactate protect against acute pancreatitis via inhibition of pancreatic and systemic inflammatory responses. Gut Microbes.

[289] Ohta T., Makino I., Okazaki M., Miyashita T., Tajima H. (2021). Intestinal Care Using L-Glutamine Supplement and Probiotics Can Induce a Strong Anti-Tumor Immune Response through the Induction of Mature Tertiary Lymphoid Structures in Pancreatic Cancer Patients Receiving Preoperative Chemotherapy. Gan Kagaku Ryoho.

[290] Asgharian A., Askari G., Esmailzade A., Feizi A., Mohammadi V. (2016). The Effect of Symbiotic Supplementation on Liver Enzymes, C-reactive Protein and Ultrasound Findings in Patients with Non-alcoholic Fatty Liver Disease: A Clinical Trial. Int. J. Prev. Med..

[291] Naseri K., Saadati S., Ashtary-Larky D., Asbaghi O., Ghaemi F., Pashayee-Khamene F., Yari Z., de Courten B. (2022). Probiotics and synbiotics supplementation improve glycemic control parameters in subjects with prediabetes and type 2 diabetes mellitus: A GRADE-assessed systematic review, meta-analysis, and meta-regression of randomized clinical trials. Pharmacol. Res..

[292] Kanazawa A., Aida M., Yoshida Y., Kaga H., Katahira T., Suzuki L., Tamaki S., Sato J., Goto H., Azuma K. (2021). Effects of Synbiotic Supplementation on Chronic Inflammation and the Gut Microbiota in Obese Patients with Type 2 Diabetes Mellitus: A Randomized Controlled Study. Nutrients.

[293] Pan Z., Mao B., Zhang Q., Tang X., Yang B., Zhao J., Cui S., Zhang H. (2022). Postbiotics Prepared Using *Lactobacillus paracasei* CCFM1224 Prevent Nonalcoholic Fatty Liver Disease by Modulating the Gut Microbiota and Liver Metabolism. Int. J. Mol. Sci..

[294] Abdelazez A., Alshehry G., Algarni E., Al Jumayi H., Abdel-Motaal H., Meng X.C. (2022). Postbiotic Gamma-Aminobutyric Acid and Camel Milk Intervention as Innovative Trends Against Hyperglycemia and Hyperlipidemia in Streptozotocin-Induced C(57)BL/6J Diabetic Mice. Front. Microbiol..

[295] Saliminejad K., Khorram Khorshid H.R., Soleymani Fard S., Ghaffari S.H. (2019). An overview of microRNAs: Biology, functions, therapeutics, and analysis methods. J. Cell Physiol..

[296] Vishnoi A., Rani S. (2017). MiRNA Biogenesis and Regulation of Diseases: An Overview. Methods Mol. Biol..

[297] Boicean A., Birsan S., Ichim C., Boeras I., Roman-Filip I., Blanca G., Bacila C., Fleaca R.S., Dura H., Roman-Filip C. (2023). Has-miR-129-5p’s Involvement in Different Disorders, from Digestive Cancer to Neurodegenerative Diseases. Biomedicines.

[298] Backes C., Meese E., Keller A. (2016). Specific miRNA Disease Biomarkers in Blood, Serum and Plasma: Challenges and Prospects. Mol. Diagn. Ther..

[299] Blasco-Baque V., Coupé B., Fabre A., Handgraaf S., Gourdy P., Arnal J.F., Courtney M., Schuster-Klein C., Guardiola B., Tercé F. (2017). Associations between hepatic miRNA expression, liver triacylglycerols and gut microbiota during metabolic adaptation to high-fat diet in mice. Diabetologia.

[300] Santos A.A., Afonso M.B., Ramiro R.S., Pires D., Pimentel M., Castro R.E., Rodrigues C.M.P. (2020). Host miRNA-21 promotes liver dysfunction by targeting small intestinal *Lactobacillus* in mice. Gut Microbes..

[301] Hochreuter M.Y., Dall M., Treebak J.T., Barrès R. (2022). MicroRNAs in non-alcoholic fatty liver disease: Progress and perspectives. Mol. Metab..

[302] Zhang T., Yang Z., Kusumanchi P., Han S., Liangpunsakul S. (2020). Critical Role of microRNA-21 in the Pathogenesis of Liver Diseases. Front. Med..

[303] Luo W., Guo S., Zhou Y., Zhao J., Wang M., Sang L., Chang B., Wang B. (2022). Hepatocellular Carcinoma: How the Gut Microbiota Contributes to Pathogenesis, Diagnosis, and Therapy. Front. Microbiol..

[304] Xie G., Wang X., Zhao A., Yan J., Chen W., Jiang R., Ji J., Huang F., Zhang Y., Lei S. (2017). Sex-dependent effects on gut microbiota regulate hepatic carcinogenic outcomes. Sci. Rep..

[305] Pant K., Yadav A.K., Gupta P., Islam R., Saraya A., Venugopal S.K. (2017). Butyrate induces ROS-mediated apoptosis by modulating miR-22/SIRT-1 pathway in hepatic cancer cells. Redox Biol..

[306] Xiang Z., Wu J., Li J., Zheng S., Wei X., Xu X. (2023). Gut Microbiota Modulation: A Viable Strategy to Address Medical Needs in Hepatocellular Carcinoma and Liver Transplantation. Engineering.

[307] Haniff H.S., Liu X., Tong Y., Meyer S.M., Knerr L., Lemurell M., Abegg D., Aikawa H., Adibekian A., Disney M.D. (2022). A structure-specific small molecule inhibits a miRNA-200 family member precursor and reverses a type 2 diabetes phenotype. Cell Chem. Biol..

[308] Yang X., Wan J., Li N., He C., Zhang Y., Ren Y., Li X., Zhu Y., Liu F., Xia L. (2022). MiR155 Disrupts the Intestinal Barrier by Inducing Intestinal Inflammation and Altering the Intestinal Microecology in Severe Acute Pancreatitis. Dig. Dis. Sci..

[309] Lv Y., Huang S. (2019). Role of non-coding RNA in pancreatic cancer. Oncol. Lett..

[310] Smolarz B., Durczyński A., Romanowicz H., Hogendorf P. (2021). The Role of microRNA in Pancreatic Cancer. Biomedicines.

[311] Wang Q., Ding H., Dong G., Xu L., Jiang F., Mao Q. (2021). Bi-direction effects between microbiome and MiRNAs in carcinogenesis. J. Cancer Res. Clin. Oncol..

[312] Shirazi M.S.R., Al-Alo K.Z.K., Al-Yasiri M.H., Lateef Z.M., Ghasemian A. (2020). Microbiome Dysbiosis and Predominant Bacterial Species as Human Cancer Biomarkers. J. Gastrointest. Cancer.

[313] Olesen S.W., Leier M.M., Alm E.J., Kahn S.A. (2018). Searching for superstool: Maximizing the therapeutic potential of FMT. Nat. Rev. Gastroenterol. Hepatol..

[314] Ding X., Li Q., Li P., Zhang T., Cui B., Ji G., Lu X., Zhang F. (2019). Long-Term Safety and Efficacy of Fecal Microbiota Transplant in Active Ulcerative Colitis. Drug Saf..

[315] Bellucci E., Chiereghin F., Pacifici F., Donadel G., De Stefano A., Malatesta G., Valente M.G., Guadagni F., Infante M., Rovella V. (2023). Novel therapeutic approaches based on the pathological role of gut dysbiosis on the link between nonalcoholic fatty liver disease and insulin resistance. Eur. Rev. Med. Pharmacol. Sci..

[316] Sastre M., Cimbalo A., Mañes J., Manyes L. (2024). Gut Microbiota and Nutrition: Strategies for the Prevention and Treatment of Type 2 Diabetes. J. Med. Food.

